# Unveiling a BRAF Signature Proficient in Accurately Capturing Oncogenic Activity and Guiding Prognostic Prediction Across Multiple Cancers

**DOI:** 10.1002/mco2.70591

**Published:** 2026-02-03

**Authors:** Kaidi Yang, Shihui Fu, Jingbing Liang, Lijuan Ding, Junhao You, Fang Li, Ye Yuan, Xiu‐Wu Bian

**Affiliations:** ^1^ Department of Oncology Hainan Hospital of Chinese People's Liberation Army General Hospital Sanya People's Republic of China; ^2^ Institute of Pathology and Southwest Cancer Center, Southwest Hospital, and The Key Laboratory of Tumor Immunopathology (Third Military Medical University) The Ministry of Education of China Chongqing People's Republic of China; ^3^ Department of Cardiology Hainan Hospital of Chinese People's Liberation Army General Hospital Sanya People's Republic of China; ^4^ Department of Health Management Center Hainan Hospital of Chinese People's Liberation Army General Hospital Sanya People's Republic of China; ^5^ Department of Medical Oncology Chongqing University Cancer Hospital Chongqing People's Republic of China; ^6^ Institute of Pathology and Southwest Cancer Center Southwest Hospital, Third Military Medical University (Army Medical University) and The Key Laboratory of Tumor Immunopathology The Ministry of Education of China Chongqing People's Republic of China

**Keywords:** BRAF mutation, BRAF oncogenic activity, DUSP6, gene signature, prognostic prediction

## Abstract

Although *BRAF* is frequently mutated across multiple cancer types, its clinical utility as a prognostic biomarker has remained inconsistent in clinical practice, likely due to additional events modulating BRAF signaling pathways. This inconsistency has driven our investigation into the broader landscape of BRAF signaling and the development of a robust molecular signature to assess BRAF‐driven oncogenic activity. To achieve this, we introduced BRAF25, a transcriptional signature designed to effectively capture BRAF oncogenic activity. Our findings reveal that 25.6% of TCGA colorectal cancer (CRC) tumors exhibit BRAF pathway activation, even in 19.4% of BRAF wild‐type (WT) cases, suggesting alternative mechanisms driving pathway activation. The BRAF‐active subtype, termed BAG‐3 (BRAF Activity Group‐3), demonstrated reduced responsiveness to chemotherapy and anti‐BRAF therapy. Notably, BRAF25 subtyping addresses the limitations of using *BRAF* mutation alone to predict patient survival. We experimentally screened and validated DUSP6 as a sensitizing target for anti‐BRAF therapy, enhancing BRAF inhibitor efficacy in CRC. Furthermore, pan‐cancer analyses implicate the BRAF25 signature in poor prognosis across diverse BRAF‐driven malignancies. In conclusion, stratifying patients by transcriptional BRAF oncogenic activity, instead of relying solely on *BRAF* mutation status, provides a more precise approach to guide clinical decision‐making and improve therapeutic outcomes.

## Graphical abstract

The BRAF25 meta‐signature effectively captures BRAF‐driven oncogenic activity. By enabling precise patient stratification with distinct mutation profiles, BRAF25 identifies BRAF‐active tumor subtypes and provides superior prognostic value compared with mutation status alone. Pan‐cancer analyses further demonstrated that elevated BRAF25 activity is associated with unfavorable clinical outcomes and reduced responsiveness to both anti‐BRAF therapies and chemotherapy, highlighting its translational significance.

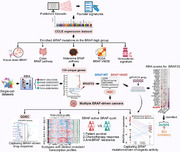

## 1 Introduction

*RAF* oncogenes encode a family of serine/threonine protein kinases that serve as central mediators of the mitogen‐activated protein kinase (MAPK) cascade, a pathway frequently co‐opted in human cancers [1, 2]. Under normal conditions, the activity of the three *RAF* isoforms (*ARAF*, *BRAF*, and *CRAF*) is regulated by GTP‐bound RAS. Point mutations in *RAF* genes, particularly within the kinase domain, can result in constitutive activation and persistent signaling independent of upstream control [3]. Such variants occur predominantly in BRAF, with the V600E substitution accounting for over 90% of cases and representing a major oncogenic driver in tumors such as cutaneous melanoma [4] and thyroid papillary cancer [5]. Numerous preclinical models have elucidated mutant *BRAF* as a key promoter of tumor initiation, progression, and cell survival [6, 7], although the predictive value of *BRAF* mutations for patient outcomes or treatment responses remains controversial.

Targeted inhibition of BRAF has demonstrated notable improvements in outcomes for patients with *BRAF*‐mutant tumors compared with conventional chemotherapy [8, 9]. However, significant challenges persist, particularly regarding the emergence of resistance. In colorectal cancer (CRC), resistance typically arises within 1 year [10], driven by transient ERK suppression followed by EGFR‐mediated reactivation of RAS and CRAF [11]. EGFR feedback effectively circumvents BRAF blockade, explaining the limited efficacy of these agents in CRC, in contrast to melanoma cells with low EGFR expression [12]. Aberrations in other components of the RAS‐RAF‐MEK‐ERK cascade may induce effects analogous to *BRAF* mutations, further explaining treatment resistance [13]. Accordingly, additional inhibition of downstream effectors can elicit deeper therapeutic responses. Notably, aberrant BRAF pathway activation also occurs in *BRAF* wild‐type (WT) tumors [14], highlighting the critical need to systematically identify all drivers of BRAF signaling through transcriptional signatures that capture oncogenic activity within this pathway.

*BRAF* mutational status alone is insufficient as a predictive biomarker for identifying patients who require specific treatment regimens. Relying solely on mutation status may obscure other critical molecular determinants (e.g., cascade mutations, epigenetic alterations, and microenvironmental regulation) that shape tumor behavior and treatment response. Current research remains limited in identifying *BRAF* mutation‐specific signatures. In ’t Veld et al. identified a 58‐gene signature distinguishing *KRAS*‐, *BRAF*‐, and *PIK3CA*‐driven CRCs [15], while Dolezal et al. developed a deep learning–based BRAF–RAS signature to classify thyroid neoplasms [16]. These studies underscore the difficulty of defining BRAF‐specific biomarkers due to extensive signaling crosstalk within the RAS–MAPK network. Comparative transcriptomic analyses between *BRAF*‐mutant and WT melanomas have identified differentially expressed genes (DEGs) [17, 18], but confounding signals from the tumor microenvironment (TME) and intratumoral heterogeneity limit their sensitivity for capturing tumor‐intrinsic BRAF activity.

In this work, we propose a framework based on BRAF oncogenic activity rather than mutation status alone to refine patient stratification. We developed a 25‐gene expression signature (BRAF25) and validated its effectiveness in both predicting *BRAF* mutation status and accurately capturing BRAF‐driven activity. By applying a machine learning‐based support vector machine (SVM) classifier to patient cohorts treated with chemotherapy or anti‐BRAF therapy, we accurately identified primary resistance in individuals with high BRAF activity. Drug sensitivity profiling in BRAF‐active cell lines further suggested rational combination therapeutic strategies. Finally, pan‐cancer analyses revealed that this framework robustly predicts oncogenic BRAF activity and is associated with survival outcomes across multiple tumor types.

## 2 Results

### 2.1 Preparing Founder Signatures Related to BRAF Pathway Signaling

Founder signatures were generated by curating BRAF‐related genes from published datasets that employed a variety of approaches to modulate BRAF pathway activity, including RNA interference, pharmacological inhibition, transgenic overexpression, and clinical case studies (Table S1). We focused on genes that were upregulated under BRAF activation and downregulated upon BRAF inhibition. To improve generalizability, we included tumor models derived from multiple organs, including skin, colon, and thyroid. Genes predominantly expressed in immune cells were coarsely removed to minimize potential confounding signals. Comparative analysis of the founder signatures revealed minimal gene overlap, highlighting the context‐specific transcriptional outputs of BRAF signaling (Figure S1A).

### 2.2 Building BRAF25 Meta‐Signature Using CCLE Expression Data

**FIGURE 1:**
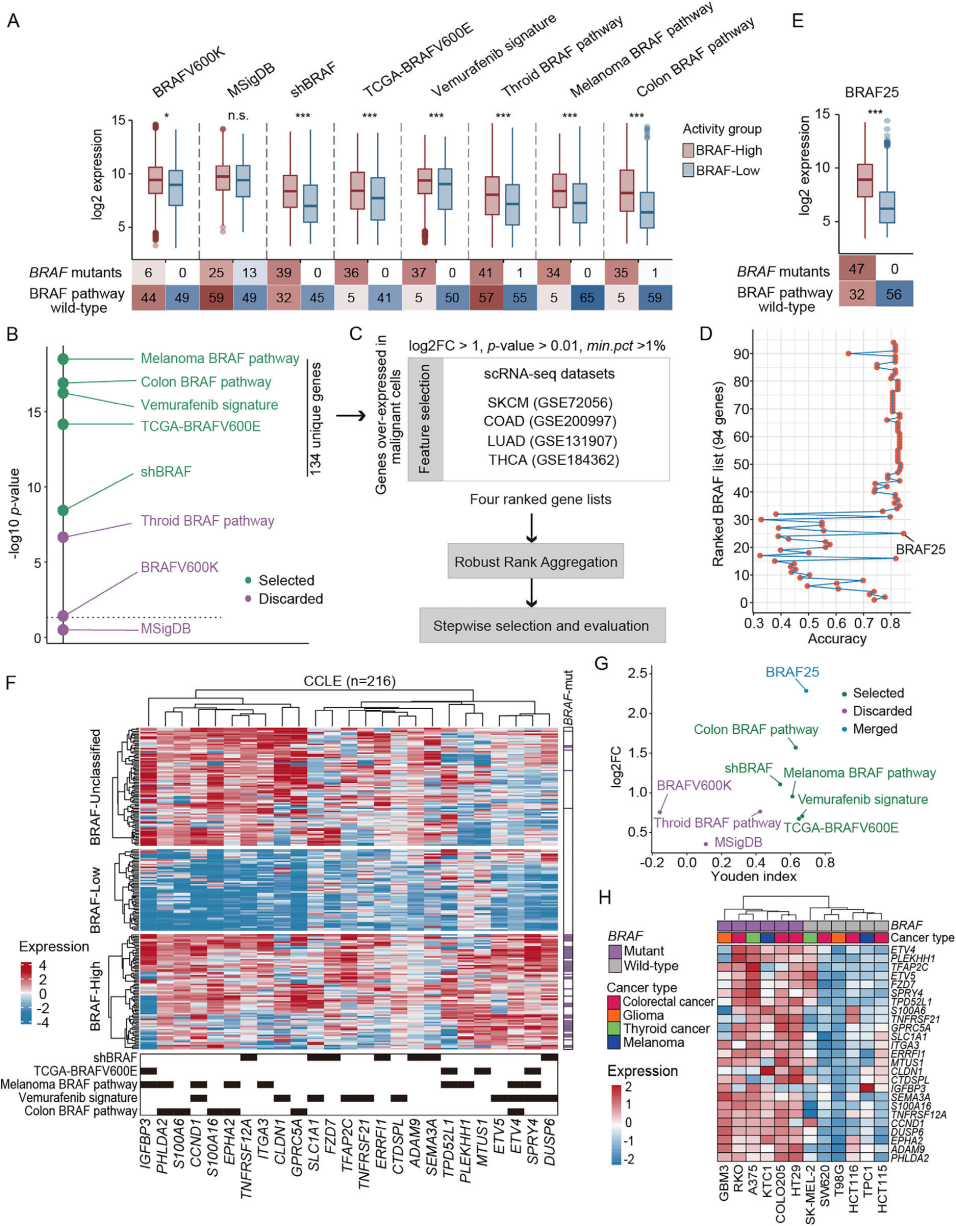
Building BRAF25 meta‐signature using CCLE expression data. (A, E) Boxplots showing the distributions of BRAF index values between BRAF‐high and BRAF‐low groups for each founder signature (A) and the BRAF25 meta‐signature (E). Boxes represent the median and interquartile range (IQR), whiskers indicate the ±1.5×IQR, and outliers beyond this range are individually labeled. Associated contingency tables display the numbers of BRAF pathway wild‐type (WT) and *BRAF* mutant cell lines in each group. WT is defined as the absence of oncogenic mutations in BRAF pathway components. Chi‐square tests were used to assess statistical independence. (B) Significance of *BRAF* mutation segregation between BRAF‐high and ‐low activity groups, as determined by chi‐square testing and denoted as −log10 *p*‐value. Selected BRAF founder signatures are marked in purple. (C) The 134 unique genes derived from selected signatures were subjected to feature selection, robust rank aggregation, and stepwise selection. (D) Line graph depicting classification accuracy (x‐axis) of gene panels with incremental sizes (adding one gene at a time, y‐axis) in correctly classifying each *BRAF*‐mutant cell line into the BRAF‐active group, or *BRAF*‐WT cell lines into the BRAF‐quiet group. (F) Heatmap of normalized expression profiles across 216 CCLE cell lines (rows) based on BRAF25 meta‐signature genes (columns). Cell lines are categorized into three groups: BRAF‐high, ‐unclassified (medium), and ‐low, according to mean BRAF index. Mutation status is indicated in the right panel, and parental signature gene origins are denoted at the bottom. (G) Scatter plot demonstrating the classification performance of different BRAF‐related signatures. On the y‐axis, the mean differential expression (log2 fold‐change) between the BRAF‐high and ‐low activity groups is shown, while the x‐axis represents the significance of *BRAF* mutation segregation, as determined by the Youden index. BRAF25 is marked in blue, and selected and discarded founder signatures are shown in blue and purple, respectively. (H) Heatmap of normalized expression profiles across 12 diverse cell lines (columns), stratified by BRAF25 genes (rows). Cell line tumor types and *BRAF* mutation status are annotated using distinct colors. ***p<0.001; **p<0.01; *p<0.05; n.s., not significant.

To ensure that founder signatures reflect BRAF oncogenic activity in tumor cells rather than stromal or immune compartments, we characterized their expression levels in cancer cell line data from the Cancer Cell Line Encyclopedia (CCLE). We prioritized tumor types with a high prevalence of *BRAF* mutations to better capture transcriptional activation driven by BRAF. A total of 315 cell lines were included, comprising 60 colorectal, 182 lung, 62 skin, and 11 thyroid cancer cell lines. To minimize confounding from parallel oncogenic pathways, we excluded cell lines carrying activating mutations in other members of the BRAF signaling pathway (*KRAS, EGFR, ERBB, FGFR1, FGFR2, FGFR3, HRAS, NRAS, ARAF, CRAF, RET*, and *KIT*). Following data normalization, we implemented a strict filtering criterion to exclude genes with low expression or variance across the founder signatures (Figure S1B). For each signature, the filtered CCLE expression matrix was stratified into three groups (BRAF‐high, BRAF‐low, or BRAF‐unclassified) based on the mean expression of the signature genes (Figure S1C). We evaluated the ability of each signature to enrich *BRAF* mutations in the BRAF‐high versus BRAF‐low groups (Figure 1A). The signatures “shBRAF,” “TCGA‐BRAFV600E,” “Colon BRAF pathway,” “Melanoma BRAF pathway,” and “Vemurafenib signature” were identified as the most significant, with potential value in quantifying BRAF oncogenic activity (Figure 1B). These five signatures were further refined through DEG analysis to identify upregulated genes in the BRAF‐high group compared with the BRAF‐low group (log2 fold‐change>2, *p*‐value < 1e‐10) (Figure S1D), yielding 134 unique genes. These genes were independently ranked based on their significance in exhibiting tumor‐dominant expression in scRNA‐seq datasets derived from colorectal, lung, skin, and thyroid cancers. The four ranked gene lists were aggregated into a single ranked gene list using the robust rank aggregation (RRA) method (Figure 1C). Using the 94‐gene panel as input for a stepwise classification strategy, a 25‐gene set with the highest discriminant performance (accuracy = 0.845) was identified as the optimal set (Figure 1D). This process established the meta‐BRAF‐activity signature, BRAF25, for pan‐cancer analysis (see Table S2 for gene details). The classification performance of BRAF25 was tested on cell line data, with 47 out of 53 *BRAF* mutant cell lines clustered in the BRAF‐high cluster, six in the unclassified cluster, and none in the BRAF‐low cluster, indicating strong discriminatory capacity (Figure 1E,F). BRAF25 demonstrated the largest activity difference between BRAF‐low and ‐high groups and achieved the highest Youden index (0.690), outperforming other BRAF founder signatures (Figure 1G). These results highlight BRAF25's ability to better capture BRAF oncogenic activity and correctly categorize *BRAF*‐mutant cell lines with activated signaling. Pathway enrichment analysis of DEGs in the BRAF‐high versus BRAF‐low groups highlighted signaling cascades such as ERK1 and ERK2 cascade, positive regulation of MAPK, and regulation of ERK1 and ERK2 as BRAF oncogenic activity‐dependent pathways (Table S3).

To validate the applicability of BRAF25, we tested its expression in 12 tumor cell lines from various organs using a customized PCR array. The mutation status of these cell lines was confirmed by immunoblotting (Figure S2A–D). After normalization and clustering, *BRAF*‐mutant and WT cell lines formed distinct groups based on BRAF25 expression (Figure 1G), a pattern that remained consistent when analysis was restricted to CRC cell lines (Figure S2E).

### 2.3 BRAF25 Facilitates Cancer Classification Characterized by Distinct Mutation and Transcription Profiles

**FIGURE 2:**
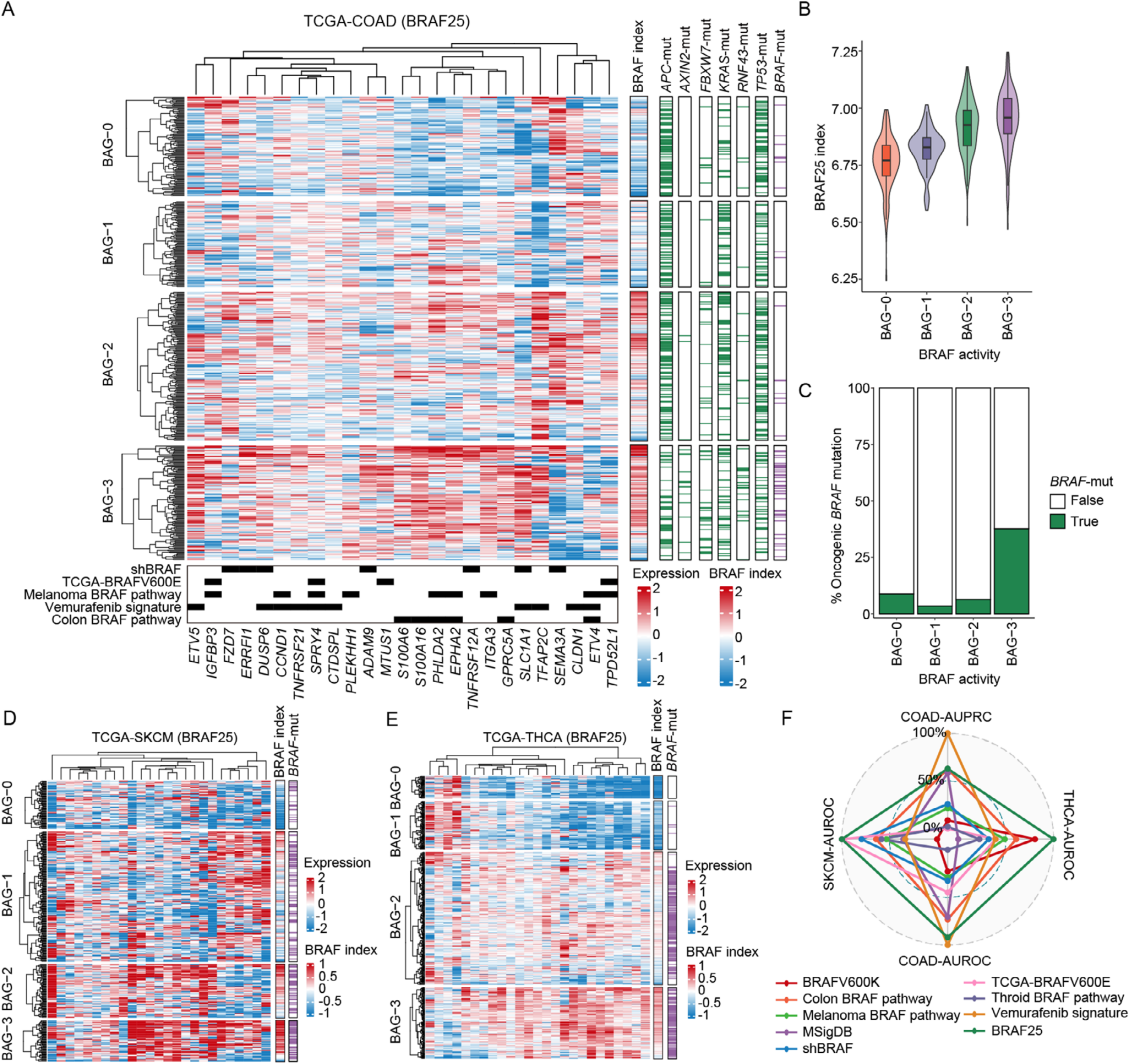
BRAF25 facilitates colon cancer classification characterized by distinct mutation and transcription profiles. (A) Heatmap of VST‐normalized RNA‐seq data from TCGA‐COAD (*n* = 465), stratified by BRAF25 signature genes (columns). Hierarchical clustering using the Ward.D2 algorithm identifies four BRAF activity groups (BAGs). The mean BRAF25 expression, termed the BRAF index (BI), is shown on the right. Genomic variants exhibiting a significant non‐random distribution (chi‐square test, *p*‐value < 0.05) across the BAGs are shown on the right. Signature gene origins from parent signatures are annotated at the bottom. (B) Violin plot showing the BRAF25 expression distribution across BAGs. (C) Bar plots showing *BRAF* mutation frequencies across BAGs. (D–E) Heatmaps of VST‐normalized TCGA‐SKCM (D) and ‐THCA (E) RNA‐seq data stratified by the BRAF25 gene (columns). (F) Radar plot displaying AUROC and AUPRC (COAD only) for BRAF25 and eight founder signatures across TCGA‐COAD, ‐THCA, and ‐SKCM. All values are normalized to a 0–1 scale for comparison.

To ensure that BRAF25 expression predominantly reflects tumor‐intrinsic BRAF activity, we investigated its expression across cell‐type compartments in single‐cell RNA sequencing (scRNA‐seq) datasets matched with cell lines. BRAF25 showed minimal expression in stromal and immune populations compared with tumor cell subsets (Figure S3). We then evaluated the broader applicability of the BRAF25 signature by applying it to TCGA colon adenocarcinoma (COAD) bulk RNA‐seq data. Hierarchical clustering analysis based on the ordered mean BRAF25 ssGSEA score (termed the BRAF index) yielded four distinct groups, designated as BRAF activity groups (BAG‐0 to BAG‐3) (Figure 2A, left panel). As expected, *BRAF*‐mutant tumors exhibited significantly higher BRAF index levels compared with *BRAF*–WT tumors (Figure S4A). BAG‐3 was designated as the BRAF‐active tumors due to its notable *BRAF* mutation rate (37.8%) and the highest average BRAF index (Figure 2B,C). Mutation patterns of frequently altered colon cancer genes (e.g., *RNF43, KRAS, FBXW7, APC, AXIN2*, and *TP53*) displayed distinct distributions across BAGs (chi‐square test, false discovery rate [FDR] < 0.01) (Figure 2A, right panel). Notably, *AXIN2*, *RNF43*, and *FBXW7* mutations closely mirrored *BRAF* alterations, peaking in BAG3. In contrast, *APC* mutations were prevalent in BAG‐0 to BAG‐2, while *KRAS* mutations were most frequent in BAG‐1, implying divergent oncogenic mechanisms (Figure 2A). Broader cross‐platform and cross‐cohort validation in CRC was further performed using CPTAC proteomics and RNA‐seq datasets, supporting the robustness and generalizability of the BRAF25 classifier (Figure S4B–C). We extended our analysis to TCGA skin cutaneous melanoma (SKCM) and thyroid carcinoma (THCA), two cancer types with the highest *BRAF* mutation rates (> 50%) across TCGA cohorts. In SKCM, *BRAF* mutation frequency increased in parallel with the BRAF index (Figure 2D), a pattern also observed in glioblastoma (GBM) (Figure S4D) and lung adenocarcinoma (LUAD) (Figure S4E). Notably, in THCA, BAG‐2 and BAG‐3 together accounted for 98.3% of all *BRAF*‐mutant tumors (Figure 2E).

We further evaluated the ability of the BRAF25 index to discriminate *BRAF*‐mutant tumors using the area under the receiver operating characteristic curve (AUROC), calculated based on the convex hull of the ROC curve (Figure 2F and Figure S4F–H). BRAF25 outperformed other founder signatures in SKCM and THCA (AUROC = 0.690 and 0.850, respectively) and ranking second in COAD (AUROC = 0.693). Given the class imbalance in the COAD cohort (*BRAF* mutation rate 14.8%), we additionally assessed performance using the area under the precision–recall curve (AUPRC). BRAF25 achieved an AUPRC of 0.25, indicating favorable discriminative performance in this context (Figure 2F and Figure S4I; see Table S4 for detailed values).

We next analyzed the distribution of *BRAF* mutational variants in TCGA‐SKCM. Among all *BRAF* mutation types, *BRAFV600E* accounted for 68.08%, while *BRAFV600K* accounted for 12.76%. Notably, *BRAFV600E* was predominantly enriched in BAG‐3 and associated with the highest BRAF activity, whereas *BRAFV600K* was more frequently observed in BAG‐0 and BAG‐1 (Figure S4J).

To determine whether the BRAF25‐based classification reflects graded pathway activity, we examined phosphorylated ERK1/2 (pT202/Y204) and MEK1 (pS217/S221) levels across BAGs using TCGA proteomic data. Consistent with the BRAF index, ERK1/2 and MEK1 phosphorylation progressively increased from BAG‐0 to BAG‐3 (Figure S4K). In contrast, upstream EGFR‐pY1068 activity showed the lowest expression in BAG‐3 (Figure S4K), suggesting feedback inhibition of upstream signaling.

### 2.4 BRAF25 Subtyping Compensates for the Limitations of BRAF Mutation in Predicting Survival and Chemotherapy Response

**FIGURE 3:**
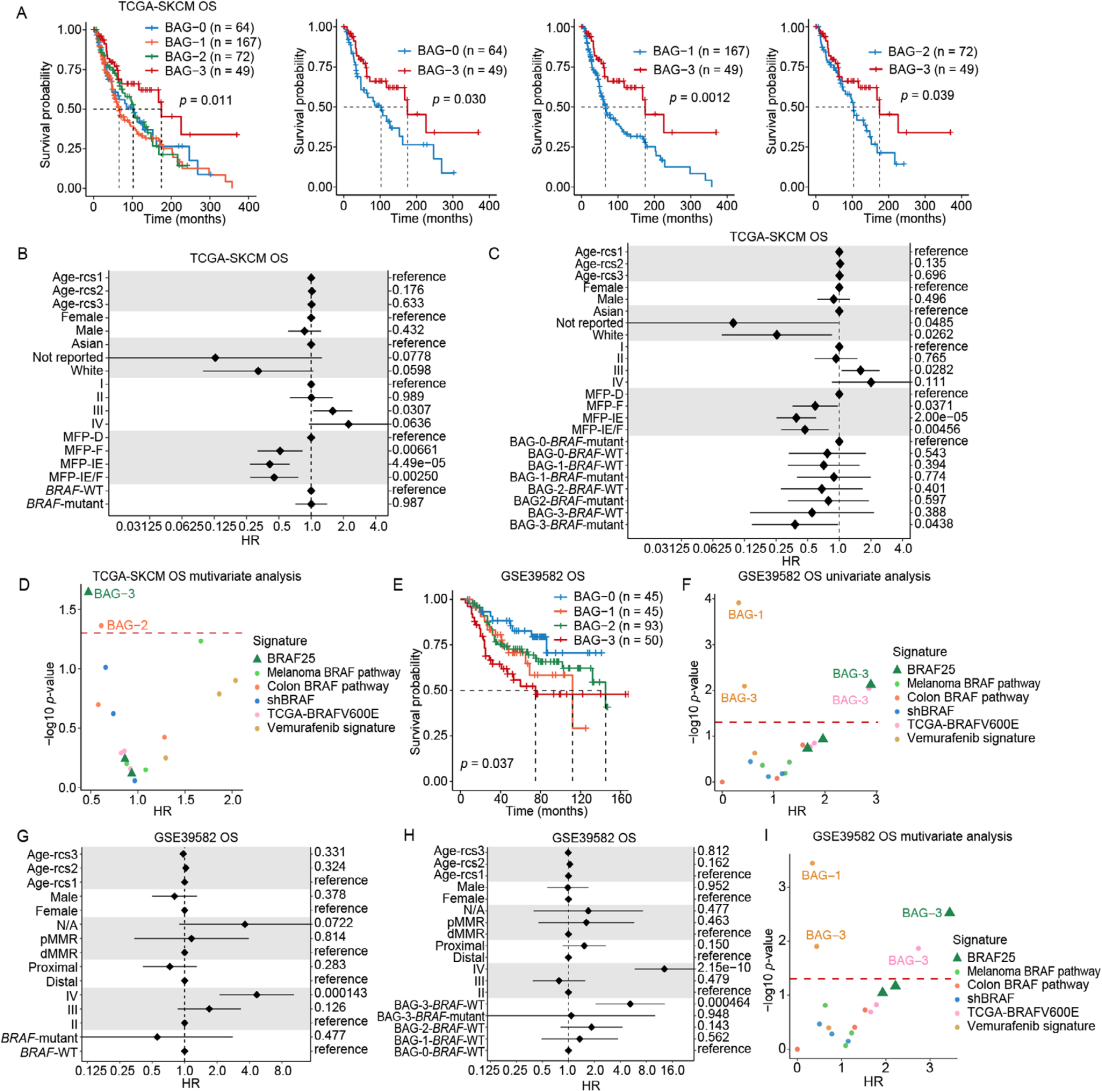
BRAF25 subtyping compensates for the limitations of *BRAF* mutation in predicting survival and chemotherapy response. (A) Kaplan–Meier plot showing overall survival (OS) across BAGs in TCGA‐SKCM (left) and a comparison of BAG‐3 versus BAG‐0 to ‐2 (right). (B) Forest plots of multivariate Cox regression for OS in TCGA‐SKCM, comparing *BRAF* mutant patients with *BRAF* ‐WT patients. (C) Forest plot of multivariate Cox regression results for OS in TCGA‐SKCM, comparing BAGs/*BRAF*‐mutant subgroups relative to the BAG‐0/*BRAF*‐mutant reference. (D) Scatter plot of multivariate Cox regression results for OS in TCGA‐SKCM. *p*‐values (on a −log10 scale) versus hazard ratios (HRs) for BAGs and founder signature‐defined groups are shown. (E) Kaplan–Meier plot for OS in adjuvant chemotherapy‐treated colon cancer patients (Laetitia cohort), stratified by BAGs. (F) Scatter plot showing the *p*‐values (on a −log10 scale) versus hazard ratios for BAGs and founder signature‐defined groups in the Laetitia's post‐chemotherapy cohort. (G) Forest plots of multivariate Cox regression for OS after chemotherapy in the Laetitia's colon cancer cohort, comparing *BRAF* mutant patients to *BRAF*‐WT patients. (H) Forest plot of multivariate Cox regression for OS after chemotherapy in the Laetitia's colon cancer cohort, comparing BAGs/*BRAF*‐mutant subgroups relative to BAG‐0/*BRAF*‐WT reference. BAG‐0 to BAG‐2 with *BRAF* mutations were excluded due to not meeting statistical testing criteria. (I) Scatter plot of multivariate Cox regression results for OS in Laetitia's colon cancer cohort. *p*‐values (on a −log10 scale) versus hazard ratios for BAGs and founder signature‐defined groups are shown. MFP, Bagaev's Molecular Functional Portrait; IE/F, immune‐enriched, fibrotic; IE, immune‐enriched, non‐fibrotic; F, fibrotic; D, immune‐depleted.

We next investigated whether the BRAF25 signature could overcome the limited prognostic value of *BRAF* mutation status in predicting patient survival. Univariate Cox proportional hazards analysis of the TCGA‐SKCM cohort showed that BAG‐3 patients had significantly improved OS compared with those in BAG‐0 to BAG‐2 (Figure 3A). Multivariate analyses confirmed that BRAF25 subtyping independently predicted survival (Figure S5A), a finding not observed for *BRAF* mutation status alone (Figure 3B). To determine whether *BRAF* mutation status could further refine the prognostic value of BRAF25 subtyping, we stratified BAGs by *BRAF* mutation status and found that *BRAF*‐mutant BAG‐3 tumors exhibited the most favorable outcomes (Figure 3C). Given the high concordance between BAG‐3 patients and those identified by the melanoma BRAF pathway signature (termed C3), we compared BAG‐3 patients with unmatched C3 patients and observed superior OS in BAG‐3 (Figure S5B), highlighting the added prognostic value of BRAF25. Multivariate comparisons across BRAF25 and its constituent founder signatures demonstrated that BAG‐3 conferred the greatest survival benefit (Figure 3D), suggesting the superior performance of BRAF25 in capturing clinically relevant BRAF oncogenic activity.

To evaluate the predictive value of BRAF25 for chemotherapy response, we applied the signature to an external Laetitia's colon cancer cohort receiving adjuvant chemotherapy. After confirming the absence of significant co‐correlation among BRAF25 genes (Figure S6A, B), we split the TCGA‐COAD BRAF25 expression matrix into training and test sets (8:2 ratio) and ranked genes by their importance in classifying the test set using a Random Forest algorithm. Iterative SVM classifiers built from ranked gene subsets identified a 23‐gene model as optimal (Figure S6C). Notably, the full 25‐gene classifier achieved the highest sensitivity and specificity for detecting BRAF‐active BAG‐3 tumors (Figure S6D) and successfully recapitulated  the enrichment of *BRAF*‐mutant case in BAG‐3 (Figure S5C). Intriguingly, BRAF‐active BAG‐3 patients exhibited the poorest OS following chemotherapy (Figure 3E), with statistical significance compared with BAG‐0 and BAG‐2 (Figure S5D). Univariate survival analysis further revealed that BAG‐3 patients showed inferior chemotherapy responses relative to those classified by other founder BRAF signatures (Figure 3F). In multivariate regression analysis, BRAF25 subtyping remained an independent predictor of OS (Figure S5E), whereas *BRAF* mutation status alone failed to predict chemotherapy response (Figure 3G). Despite limited statistical power due to small subgroup sizes, BAG‐3 tumors with *BRAF*‐WT status exhibited the lowest chemotherapy responsiveness (Figure 3H), suggesting that transcriptional BRAF activity operates independently of mutational status. Comparative analyses confirmed that BAG‐3, defined by BRAF25, outperformed other BRAF‐related signatures in predicting chemoresistance (Figure 3I). Collectively, these findings demonstrate that BRAF oncogenic activity provides superior predictive power for chemotherapy response, whereas *BRAF* mutation status alone is insufficient. The 25‐gene SVM classifier represents a robust and clinically actionable surrogate for BAG reproducibility in precision oncology.

### 2.5 BRAF25 Subtyping Predicts Resistance to Anti‐BRAF Therapy in Melanoma Patients

**FIGURE 4:**
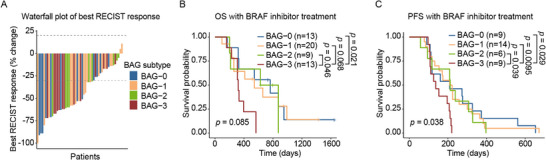
BRAF25 subtyping predicts anti‐BRAF therapy resistance for melanoma patients. (A) Waterfall plot of best overall responses (RECIST) among evaluable patients (*n* = 52) stratified by BAG‐0 to BAG‐3. Dashed lines denote the 20% tumor increase and the 30% tumor reduction thresholds. (B, C) Kaplan–Meier curves showing OS (B) and progression‐free survival (PFS) (C) for BAG‐3 versus BAG‐0 to ‐2. BRAFi, BRAF inhibitor.

Despite substantial progress in targeted therapy for *BRAF*‐mutant melanoma, acquired drug resistance remains a major clinical challenge. To determine whether BRAF25‐based subtyping could predict anti‐BRAF therapeutic response, we applied an SVM classifier trained on the TCGA‐SKCM dataset to three independent melanoma cohorts and successfully recapitulated the BAG classification. Clinical efficacy data revealed that although BAG‐3 melanomas initially responded favorably to anti‐BRAF therapy (Figure 4A), they ultimately exhibited the poorest OS and PFS (Figure 4B,C). Statistically significant differences were observed when compared with BAG‐0 (PFS: *p*‐value 0.029; OS: *p*‐value 0.021), BAG‐1 (PFS: *p*‐value 0.0095; OS: *p*‐value 0.068), and BAG‐2 (PFS: *p*‐value 0.039; OS: *p*‐value 0.046).

### 2.6 BRAF25 can Better Capture BRAF‐Driven Drug Response

**FIGURE 5:**
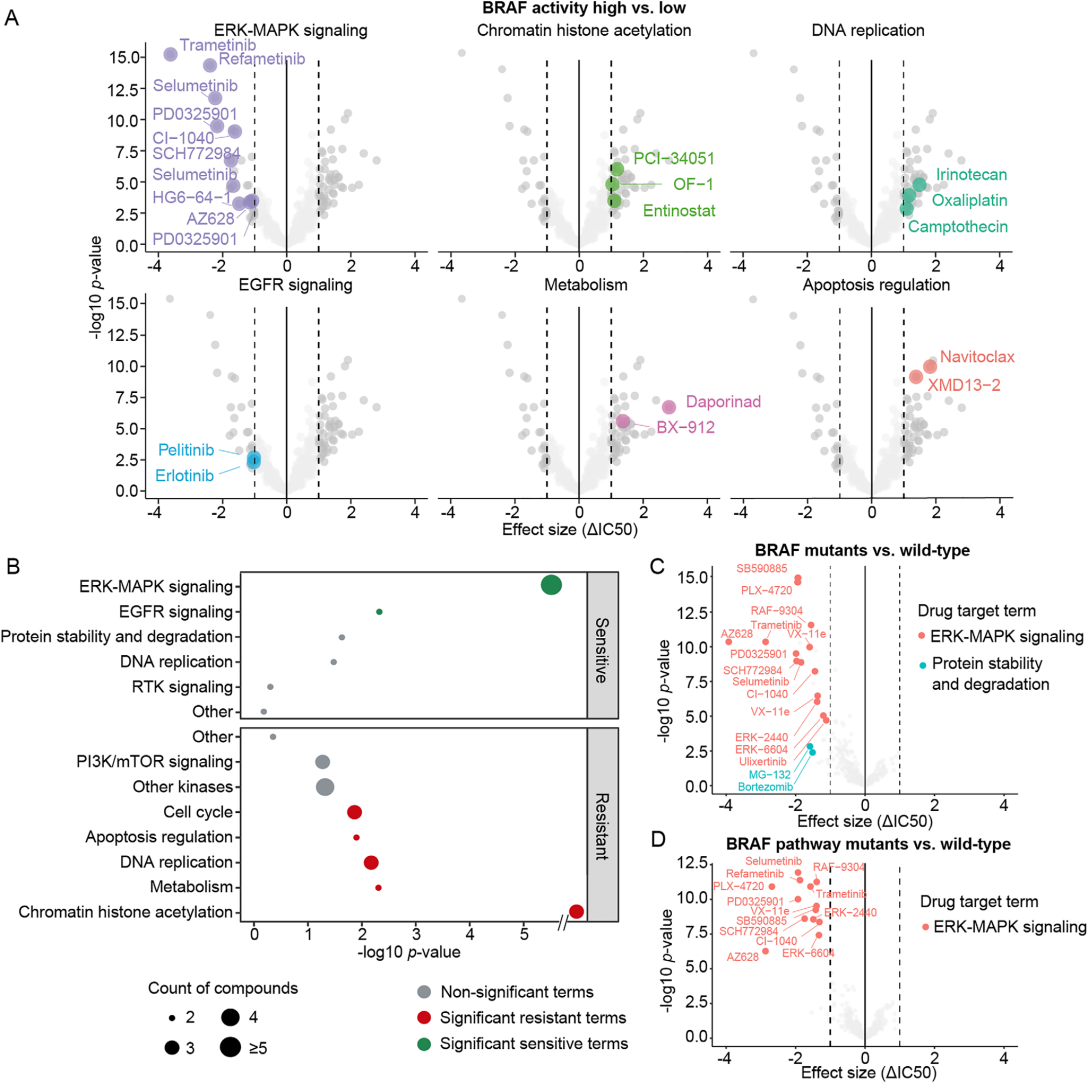
BRAF25 can better capture BRAF‐driven drug response. (A) Volcano plots showing the significance (y‐axis) and differential response (x‐axis) in half‐maximal inhibitory concentration (IC50) values across the GDSC compound library between BRAF25‐high and ‐low activity CCLE cell lines, faceted by enriched drug target categories (FDR < 0.05, hypergeometric test). Drugs with log2 fold‐change > 1 and FDR < 0.05 (controlled by the Benjamini–Hochberg correction) are depicted in dark gray and highlighted with distinct colors when lying within a specific drug target term. (B) Enrichment plot showing drug targeting annotations associated with sensitive (green) and resistant (red) drugs in BRAF25‐high versus ‐low activity groups (hypergeometric test, FDR < 0.05). Circle size denotes the number of associated drugs. (C, D) Volcano plots showing the significance (y‐axis) and differential response (x‐axis) in IC50 between (C) *BRAF* mutant versus WT and (D) BRAF pathway mutant versus WT cell lines. Enriched drug target terms with statistical significance (log2 fold‐change > 1, FDR < 0.05) are highlighted in different colors. One outlier drug targeting ERK‐MAPK is omitted for clarity.

The capacity of BRAF25 to capture BRAF oncogenic activity‐driven drug response was assessed using drug sensitivity data from the Genomics of Drug Sensitivity in Cancer (GDSC) project. Dabrafenib, an FDA‐approved BRAF inhibitor for *BRAFV600E* tumors, emerged as the most effective drug across *BRAF*‐mutant tumor cell lines, confirming the reliability of the drug sensitivity data (Figure S7A). Subsequently, we screened for drugs exhibiting differential sensitivity between the two BRAF activity groups (FDR < 0.05, log2 (ΔIC50) > 1 or < −1). Categorization of drug candidates by their annotated targets revealed that BRAF‐high cell lines were preferentially sensitive to drugs targeting the ERK‐MAPK and EGFR signaling pathways (Figure 5A,B). Notably, previous studies have demonstrated that combining EGFR inhibitors can overcome resistance to BRAF inhibitors [19]. In contrast, BRAF‐high cell lines exhibited resistance to drugs targeting chromatin histone acetylation, metabolism, DNA replication, apoptosis regulation, and cell cycle (Figure 5A,B).

Further comparison of drug sensitivity in the context of *BRAF* mutation (Figure 5C) and broader BRAF pathway mutation dependence (Figure 5D) showed consistent enrichment of ERK‐MAPK signaling. Most drug candidates identified through the BRAF activity‐based comparison overlapped with those enriched in *BRAF* mutation‐ or BRAF pathway mutation‐dependent contexts (Figure S7B and Table S5). These findings suggest that the BRAF25 signature effectively captures BRAF‐driven drug response, recapitulating the effects observed with *BRAF* mutation or BRAF pathway alterations. The enriched drug target categories may inform combination strategies to overcome resistance to BRAF inhibitors.

### 2.7 DUSP6 Identified as a Promising Therapeutic Target for Combination Therapy

**FIGURE 6:**
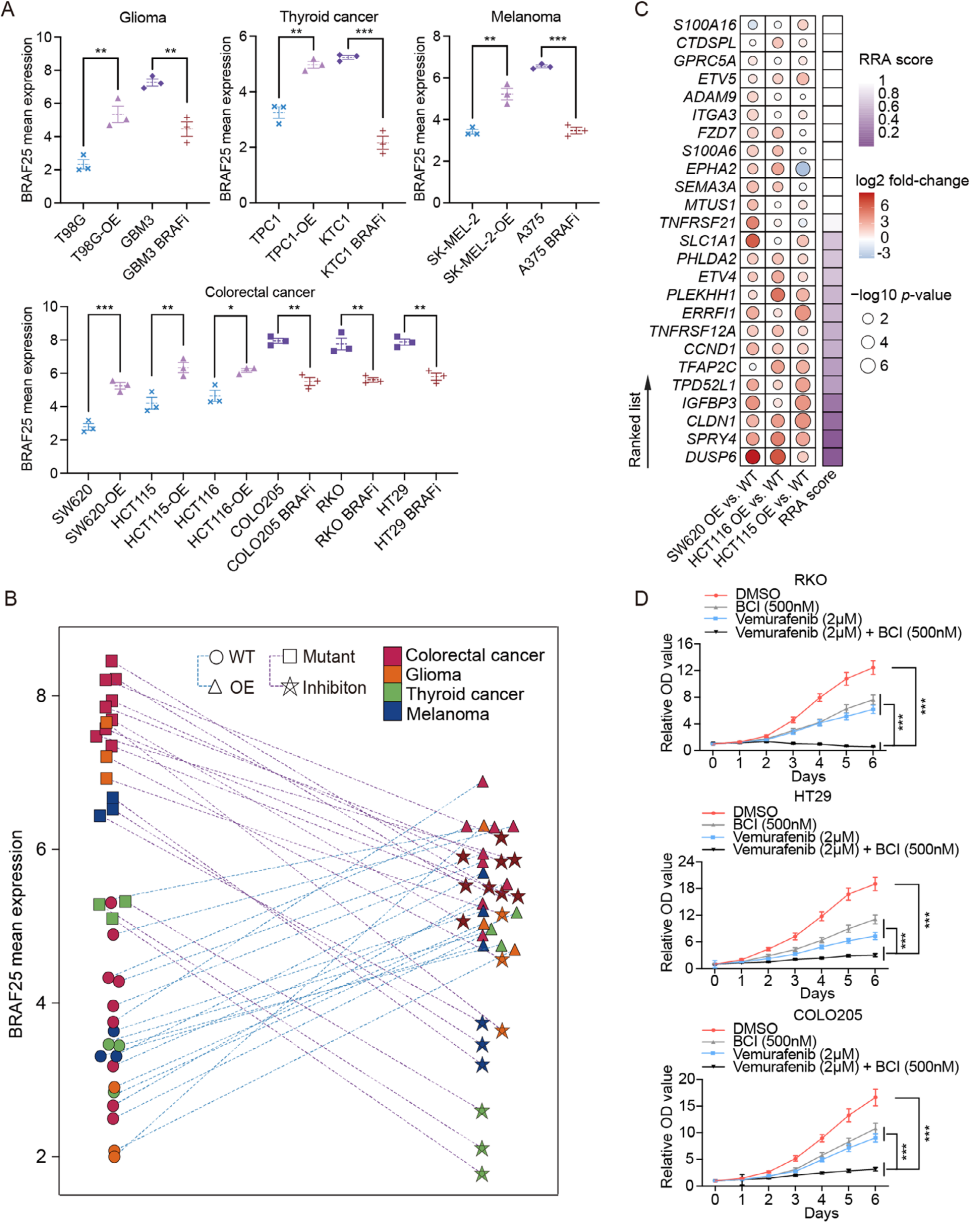
*DUSP6* identified as a promising therapeutic target for combination therapy. (A) Mean expression levels of the BRAF25 signature across distinct cell types in control, BRAFV600E overexpression (OE), and anti‐BRAF treatment groups. Error bars represent mean ± SEM (*n* = 3), and group means are indicated by dashed lines. (B) Paired comparison of mean BRAF expression between control and treatment conditions. Each pair represents three independent pre‐ and post‐treatment samples per subgroup, connected by dashed lines. Cancer types are color‐coded, while treatment modality (OE or anti‐BRAF inhibitor) and *BRAF* mutation status (WT or mutant) are denoted by different symbols. (C) Heatmap of robust rank aggregation (RRA) scores for BRAF25 signature genes (rightmost column) across individual colorectal cancer cell lines. Corresponding fold changes and adjusted *p*‐values are visualized on the left using dot plots, where dot color indicates log2 fold‐change level and dot size represents −log10 adjusted *p*‐value. (D) Relative optical density (OD) values from CCK8 assays in RKO, HT29, and COLO25 cells treated with BCI or Vemurafenib, either alone or in combination. ***p<0.001; **p<0.01; *p<0.05.

Intriguingly, treating *BRAF*‐mutant cells with the BRAF inhibitor Vemurafenib led to a broadly consistent downregulation of BRAF25 signature genes across glioma, thyroid cancer, melanoma, and CRC cell lines, whereas *BRAFV600E* overexpression resulted in marked upregulation of most signature genes (Figure S8). Notably, mean BRAF25 expression exhibited a coordinated and uniform pattern in response to these perturbations across diverse tumor types, supporting the robustness of BRAF25 in capturing oncogenic BRAF signaling activity (Figure 6A,B). To identify the most predictive gene for BRAF activity and guide combination therapy, we applied the RRA method to integrate gene expression changes from *BRAFV600E* overexpression models across three *BRAF*‐mutant CRC lines. Among the BRAF25 genes, *DUSP6* emerged as the most consistent and pronounced alteration in expression (Figure 6C). Given that DUSP6 has a known small‐molecule inhibitor, BCI [20], we selected it as a co‐target alongside BRAF inhibition. Half‐maximal inhibitory concentration (IC50) analysis confirmed that *BRAF*‐mutant CRC cell lines were more sensitive to BCI than WT cells (Figure S9A–C). Although BCI monotherapy only modestly suppressed growth relative to Vemurafenib alone, the combination of BCI and Vemurafenib significantly enhanced anti‐tumor efficacy in all three *BRAF*‐mutant cell lines (Figure 6D), indicating a synergistic sensitization effect. Collectively, these results highlight *DUSP6* as a promising therapeutic target for potentiating anti‐BRAF therapy.

### 2.8 BRAF25 Predicts Patient Survival in Multiple BRAF‐Driven Cancers

**FIGURE 7:**
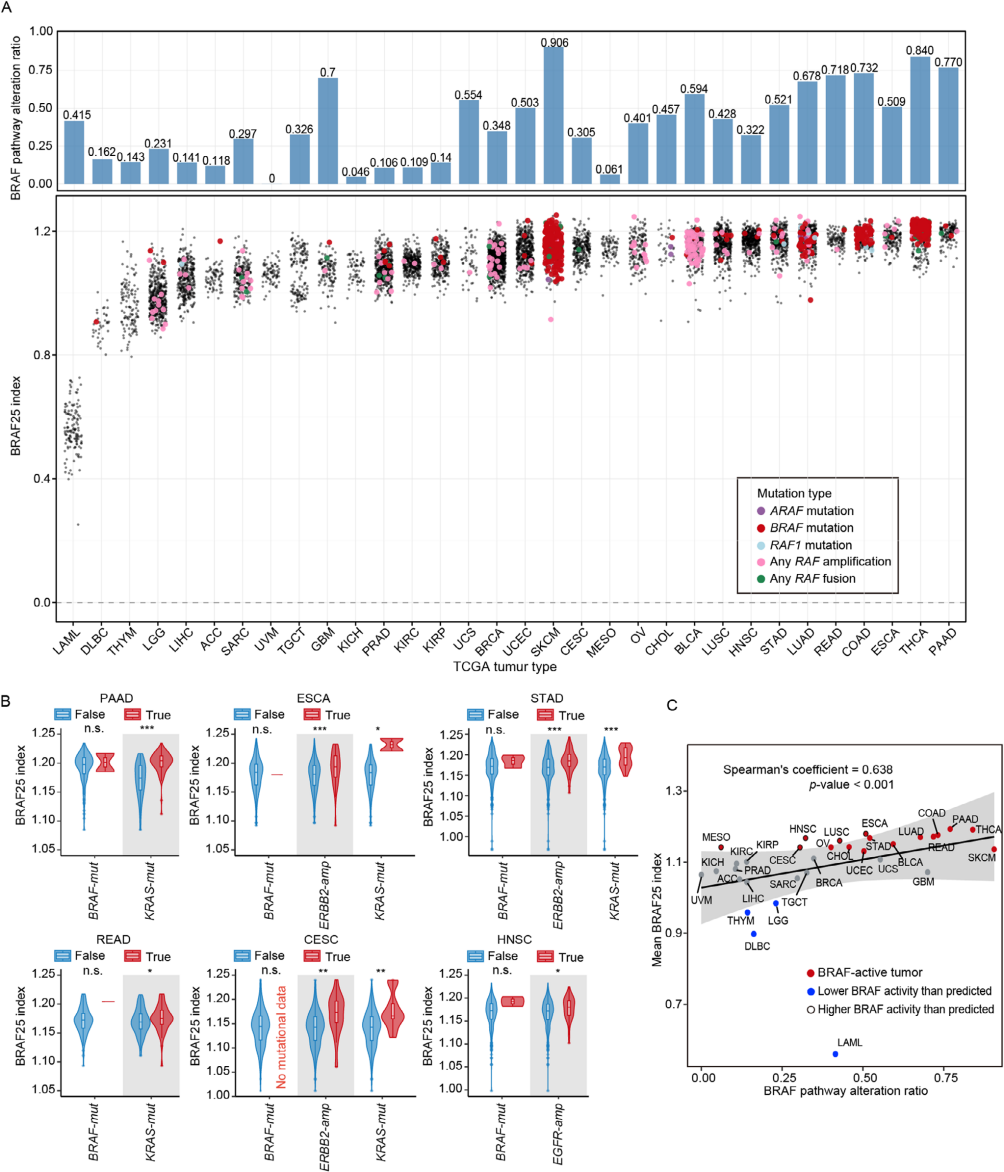
BRAF25 predicts patient survival in multiple BRAF‐driven cancers. (A) Aggregated scatter plots showing the BRAF index value (x‐axis) for each patient across TCGA pan‐cancer cohorts, with specific *RAF* alterations highlighted in distinct colors. The upper panel displays a bar chart showing the BRAF pathway member alteration ratio. (B) Violin plots showing the distribution of BRAF index values in TCGA‐LGG, ‐LUAD, and ‐LUSC cohorts, stratified by *BRAF* and key BRAF pathway members (*KRAS, ERBB2, EGFR*). Boxes within each violin indicate the median and interquartile range (IQR), with whiskers extending to ±1.5 × IQR. Outliers are explicitly marked. (C) Scatter plot assessing the relationship between the BRAF pathway mutation ratio (x‐axis) and the mean BRAF25 index (y‐axis) across the TCGA cancer cohorts. The correlation was fitted by a blue linear regression line, accompanied by a 99% confidence interval (CI) represented by the grey ribbon. Spearman correlation results are reported. BRAF‐active tumors (marked in red) above the confidence interval are outlined in black, while those below are labeled in blue. ***p<0.001; **p<0.01; *p<0.05; n.s., not significant.

The contribution of *BRAF* mutations to tumor initiation and progression varies across cancer types, with melanoma, thyroid cancer, and colon cancer showing relatively high *BRAF* mutation frequencies, whereas kidney and liver cancers exhibit low frequencies. To assess whether the BRAF25 signature captures these variations, we quantified the BRAF index across all TCGA solid tumor cohorts (Figure 7A). Based on the density distribution of mean BRAF index values, cancers were stratified into two distinct groups, termed the BRAF‐active and BRAF‐quiet groups. The BRAF‐active group included tumor types with high *BRAF* mutation rates, including THCA (62.50%), SKCM (53.74%), COAD (16.90%), bladder urothelial carcinoma (BLCA, 12.97%), LUAD (9.79%), and cholangiocarcinoma (CHOL, 8.57%) (Figure S10A). Interestingly, several cancers with low *BRAF* mutation frequencies were also classified into the BRAF‐active group. These included pancreatic adenocarcinoma (PAAD, 2.63%), esophageal carcinoma (ESCA, 4.14%), rectum adenocarcinoma (READ, 2.56%), head and neck squamous cell carcinoma (HNSC, 1.02%), stomach adenocarcinoma (STAD, 3.68%), and cervical squamous cell carcinoma and endocervical adenocarcinoma (CESC, 0.37%). In these cases, elevated BRAF activity was largely driven by mutations in upstream pathway members, such as *EGFR*, *ERBB2*, and *KRAS*, rather than *BRAF* itself (Figure 7B).

To further explore this observation, we analyzed the pan‐cancer correlation between the BRAF pathway alteration ratio and the mean BRAF index. Tumors were considered BRAF pathway‐altered when at least one genetic alteration was detected within the extended BRAF signaling cascade (RTK‐RAS‐RAF‐MEK‐ERK). A significant positive correlation was observed across cancer types (Spearman coefficient = 0.610, *p*<0.001) (Figure 7C). Notably, the mean BRAF index values in lung squamous cell carcinoma (LUSC), mesothelioma (MESO), HNSC, ESCA, and CESC exceeded that predicted by their BRAF pathway alteration ratios (Figure 7C), suggesting alternative mechanisms of BRAF pathway activation beyond detected genetic alterations. Conversely, brain lower grade glioma (LGG), thymoma (THYM), acute myeloid leukemia (LAML), and lymphoid neoplasm diffuse large B‐cell lymphoma (DLBC) exhibited lower BRAF activity than expected. *RAS* oncogene mutations are prevalent in hematopoietic tumors [21]. However, because BRAF25 excluded immune components, the high prevalence of *RAS* mutations (mostly *KRAS* and *HRAS*) did not correlate with BRAF activity in THYM and LAML. In LGG, we observed a significant association between *BRAF* mutant status and elevated BRAF index (Figure S10B), excluding the possibility that *BRAF* alterations in gliomas are nonfunctional. Although 45.6% of BRAF pathway alterations in LGG were due to *EGFR* amplification, this event may not activate downstream RAS‐MAPK signaling as effectively as *EGFR* mutations, potentially explaining the discrepancy between mutation burden and observed pathway activity.

To further validate that BRAF25 reflects pan‐RAF (ARAF, BRAF, RAF1) activity, we compared the mean BRAF index between pan‐RAF mutated and WT tumors across cancer cohorts. A significant positive association was observed in COAD, SKCM, and THCA (Figure S10C). In contrast, LUAD and LUSC showed no correlation, likely due to frequent *EGFR* and *KRAS* mutations driving BRAF signaling in non‐small cell lung cancer (NSCLC) (Figure S10C). Similar trends were observed when analyzing *BRAF* mutations alone (Figure S10B).

**FIGURE 8:**
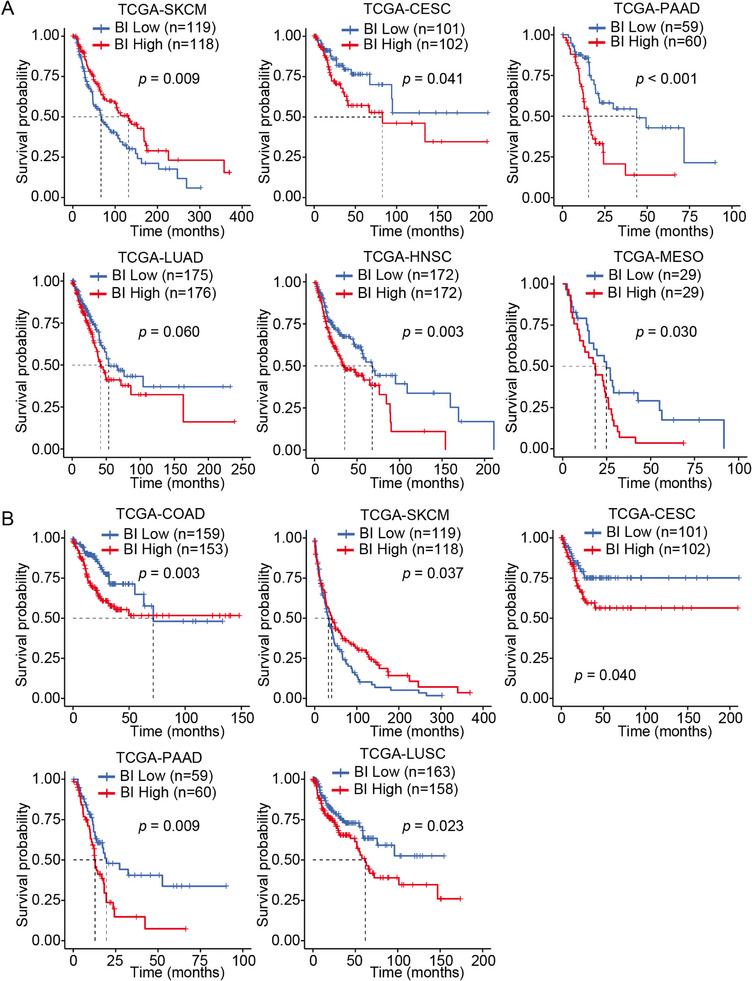
BRAF25 expression predicts patient survival in multiple BRAF‐driven cancers. (A, B) Kaplan–Meier plots of OS (A) and PFS (B) in patients with BRAF‐driven cancers, stratified into BRAF index‐high and ‐low groups. Each cohort was divided into three groups based on the BRAF25 index.

To evaluate the prognostic potential of the BRAF25 signature, patients in BRAF‐active tumor types were stratified into tertiles based on the BRAF index. A higher BRAF index was significantly associated with poorer overall survival (OS) in CESC, LUAD, HNSC, MESO, and PAAD (Figure 8A). Moreover, significant differences in progression‐free survival (PFS) were observed between high‐ and low‐BRAF activity groups in COAD, SKCM, CESC, PAAD, and LUSC (Figure 8B), reinforcing the potential of BRAF25 as a prognostic indicator in BRAF‐driven cancers.

## 3 Discussion

Mutations in the *BRAF* oncogene result in aberrant kinase activation, which drives cancer initiation, progression, and therapy resistance, as evidenced by preclinical studies [22, 23, 24, 25]. However, the prognostic impact of *BRAF* mutations remains controversial, especially in BRAF‐driven malignancies such as melanoma and CRC. For instance, Geisler et al. reported a 23% increase in relative mortality risk in melanomas harboring *BRAF* mutations, although considerable heterogeneity across studies has hindered definitive conclusions [26]. Importantly, BRAF signaling can be activated independently of mutation through upstream signals. We therefore hypothesize that *BRAF*–WT tumors may display downstream pathway activation comparable to their mutant counterparts. Given that single‐mutation detection fails to comprehensively reflect the functional state of BRAF signaling, a multi‐gene expression‐based strategy to quantify pathway activation could provide significant translational and mechanistic insight.

Current efforts to define tumor‐intrinsic transcriptional signatures for *BRAF* mutations are hampered by signaling crosstalk within the RAS–MAPK pathway and interference from the TME [15, 16, 17, 18]. Our BRAF25 meta‐signature overcomes these challenges by integrating multiple founder signatures and focusing on tumor‐intrinsic genes associated with oncogenic potential and functional activity. By combining data from controlled transgenic mouse models and human cancer cell lines while stringently filtering out TME signals, BRAF25 enables robust, tumor‐type‐independent assessment of BRAF signaling. Applying this framework across multiple BRAF‐driven cancer models ensured generalizability and pan‐cancer consistency.

Our pan‐cancer analysis revealed a strong correlation between graded BRAF activity levels and BRAF pathway mutation rates. Because genetic alterations often co‐occur across multiple pathway members such as *RAS* and *EGFR*, simple mutation‐based stratification fails to capture pathway dynamics. Consistent with prior reports that over 20% of *BRAF*–WT colorectal tumors resemble *BRAF*‐mutant expression patterns [14], we observed measurable BRAF pathway activation in *EGFR/HER2/KRAS*‐mutant tumors, and even in mutation‐negative cases, suggesting the existence of yet‐uncharacterized regulatory elements or feedback circuits that sustain oncogenic signaling independently of canonical driver mutations. Indirect mechanisms—including epigenetic alterations [27], microenvironmental regulation, autocrine growth factor secretion [28], negative feedback loop regulation [29], and metabolic stress [30]—likely contribute to this activity. Given that upstream regulatory changes are typically reflected in transcriptional variability, our approach is well‐suited to detect them without requiring full genomic or epigenetic characterization.

Functionally, BRAF25 demonstrated superior predictive capacity for both prognosis and therapeutic response compared with mutation status alone. Unexpectedly, in *BRAF*‐mutant melanoma, high BRAF25‐expressing tumors exhibited initial sensitivity but subsequently developed resistance, indicating that the signature captures both oncogenic intensity and functional implications under therapeutic pressure. It has been reported that drug resistance inevitably occurs with the use of BRAF inhibitors, even in combination with MEK inhibitors [31]. The mechanism underlying the phenomenon is the reactivation of the MAPK pathway under drug pressure, driven by RTK‐mediated activation of alternative signaling pathways and loss of feedback inhibition [11, 32, 33]. Our finding of BRAF25‐high patients responding poorly to BRAF inhibitors indicates the possible link between BRAF25 genes and MAPK reactivation‐mediated resistance, consistent with previous studies [34, 35, 36]. Mechanistically, we identified DUSP6, a negative regulator of the MAPK pathway [37, 38], as a critical hub within the BRAF25 signature. Prior studies show that DUSP6 inactivation selectively impairs the growth of *NRAS*‐ and *BRAF*‐mutant cells by inducing MAPK pathway hyperactivation. Furthermore, resistance to MAPKi ultimately induces a state of hypersensitivity to genetic knockout of DUSP4/6 [39]. Moreover, DUSP6 has been shown to promote CRC progression and is associated with poor outcomes [40]. Consistent with this, we demonstrated that pharmacologic inhibition of DUSP6 using BCI enhances the sensitivity of colon cancer cells to BRAF inhibition, nominating DUSP6 as a promising therapeutic target warranting further elucidation for its functional mechanisms in resistant models and in vivo.

Our 25‐gene classification framework paves the way for broader translational application through a customized PCR array. This assay leverages an accessible and cost‐effective qPCR platform, avoiding reliance on high‐throughput sequencing. With high‐throughput capacity, rapid turnaround, and strong standardization, it is well‐suited for clinical implementation. When combined with conventional *BRAF* mutation testing, the panel enables precise identification of tumors with bona fide BRAF pathway activation, enhancing the functional resolution of diagnostic strategies.

Despite its promising nature, the BRAF25 signature has several limitations. It relies on high‐quality RNA, which may restrict use in degraded or low‐input samples such as FFPE biopsies. Functional validation of individual genes remains incomplete, raising mechanistic uncertainties. Furthermore, the absence of standardized scoring thresholds and prospective clinical validation raises concerns regarding inter‐cohort reproducibility and clinical interpretability. Thus, BRAF25 should be regarded as a complement rather than a replacement for genotypic testing in clinical decision‐making.

Systematic analyses demonstrate that BRAF25 signature effectively captures BRAF‐driven oncogenic activity and exhibits superior predictive accuracy over mutational status. We further established its utility in stratifying prognostic subgroups across diverse TCGA cohorts, including patients treated with chemotherapy or BRAF‐targeted therapies. Notably, combinatorial targeting of DUSP6, a functional hub gene within BRAF25, may enhance anti‐BRAF treatment efficacy and guide therapeutic prioritization. Together, this integrated approach has the potential to improve clinical decision‐making in BRAF‐driven malignancies, effectively linking molecular profiling with precision oncology.

## 4 Materials and Methods

### 4.1 Acquisition of the Founder Gene Sets

The gene sets used in this study were curated from previously published resources. The BRAFV600K signature contained 309 genes upregulated following transgenic expression of *BRAFV600K* in the mouse intestinal epithelium during the tumor initiation phase [41]. The shBRAF signature consisted of 211 genes downregulated following doxycycline‐inducible knockdown of BRAF in A375 melanoma cells compared with untreated controls [42]. Three BRAF pathway signatures (melanoma, thyroid, and colon) were derived from transgenic mouse models in which *BRAFV600E* was expressed in melanocytes, thyroid epithelial cells, or intestinal epithelium under tissue‐specific promoters, respectively, to elucidate the oncogenic role of *BRAFV600E* across different tissue contexts [43, 44, 45]. The TCGA‐BRAFV600E signature was generated by identifying the top 200 most significant DEGs between *BRAF*‐mutant and WT samples in TCGA melanoma cohort [17]. Additionally, the Vemurafenib response signature, consisting of 167 genes, captured transcriptional changes induced by Vemurafenib treatment [46]. *The REACTOME_SIGNALING_BY_MODERATE_KINASE_ACTIVITY_BRAF_MUTANTS* signature was retrieved from the Molecular Signatures Database (MSigDB; https://www.gsea‐msigdb.org/gsea/msigdb) and comprised 45 genes representing moderate kinase activity associated with *BRAF* mutants.

### 4.2 CCLE Analysis

We accessed the CCLE microarray expression and mutation data via the legacy repository (https://data.broadinstitute.org/ccle_legacy_data/). Cell lines were first classified by tissue of origin, and those with potentially misannotated cancer types were excluded based on the analysis by Salvadores et al. [47]. Based on *BRAF* mutation prevalence, 315 lung, colon, skin, and thyroid cancer‐derived cell lines were selected for downstream analyses. These cell lines were classified as *BRAF* mutant, *BRAF* pathway mutant, or *BRAF* pathway mutation‐WT according to their mutational profiles. Following the removal of cell lines harboring mutations in BRAF pathway members other than *BRAF* itself, 216 tumor cell lines (127 lung, 54 skin, 27 colon, and 8 thyroid) were retained. To identify signature genes with tumor‐cell dominant expression, we performed loess regression of the log coefficient of variation against mean log2 gene expression across all genes. Signature genes with positive residuals from the fitted regression line and mean log2 expression exceeding 6 were retained.

Using the *hclust* function with the Ward.D2 algorithm, we performed hierarchical clustering of cell line transcriptomes for each filtered signature. Clusters were categorized as high, low, or unclassified based on the ranked average expression of signature genes. The ability of each signature to distinguish *BRAF* mutation‐enriched clusters was assessed using chi‐square tests. The five most discriminative signatures were selected for further refinement.

DEG analysis between the high and low clusters was performed using the *limma* package. Genes with a fold change > 2 and an FDR ＜ 0.01 were considered as key drivers contributing to molecular classification. Significant genes from each signature were merged into a unified 134‐gene BRAF meta‐signature for downstream analysis. Gene ontology (GO) enrichment analysis for biological process terms was conducted using the *clusterProfiler* (3.12.0) package, with an FDR threshold of < 0.01.

### 4.3 RRA and Stepwise Classification Analysis

To integrate the four ranked gene lists, we performed RRA using the *RobustRankAggreg* R package. This method incorporates positional information across ranked lists and assigns statistical significance to the aggregated results under a theoretical null model [48]. The resulting list of 94 aggregated genes was subjected to stepwise selection to determine the optimal panel size for classification. Starting from the top‐ranked gene, we iteratively expanded the classifier by adding one gene at a time and evaluated classification performance at each step. Model performance was assessed based on the ability to correctly categorize *BRAF*‐mutant samples as BRAF‐active and BRAF WT samples as BRAF‐quiet. The gene panel that yielded the highest classification accuracy was selected as the candidate BRAF signature.

Accuracy is calculated using the following formula:

$$\mathrm{Accuracy}\;=\frac{\mathrm{TP}+\mathrm{TN}}{\mathrm{TP}\;+\;\mathrm{TN}+\;\mathrm{FP}\;+\mathrm{FN}}$$

where:

- TP (True Positive): BRAF‐mutant cases correctly classified as BRAF‐active.
- TN (True Negative): BRAF‐WT cases correctly classified as BRAF‐quiet.
- FP (False Positive): BRAF‐WT cases incorrectly classified as BRAF‐active
- FN (False Negative): BRAF‐mutant cases incorrectly classified as BRAF‐quiet.

The Youden index was calculated as: Sensitivity+Specificity−1 With:

$$\mathrm{Sensitivity}\;=\frac{\mathrm{TP}}{\mathrm{TP}\;+\mathrm{FN}};\mathrm{Specificity}\;=\frac{\mathrm{TN}}{\mathrm{TN}\;+\mathrm{FP}}$$

### 4.4 Drug Sensitivity Analysis in CCLE Cell Lines

To assess drug sensitivity, IC50 data for 367 compounds from GDSC1 and 198 compounds from GDSC2 database were obtained, covering 24 distinct signaling pathways. Variance stabilizing transformation (VST)‐normalized RNA‐seq data were stratified into high and low BRAF activity clusters based on BRAF25 expression. Differences in drug response (IC50) between these two clusters were assessed using a linear model adjusted for *BRAF* mutation status, with significance determined using the Benjamini–Hochberg procedure (FDR < 0.05). Enriched drug target pathways among significantly associated compounds were identified using a hypergeometric test based on the “TARGET_CATEGORY” annotation (FDR < 0.05). By integrating GDSC drug response profiles with corresponding genotype information, we further identified compounds exhibiting significantly differential responses in cell lines harboring oncogenic *BRAF* or BRAF pathway mutations compared with *BRAF*‐WT cell lines. The more significant drugs/terms from the comparison between GDSC1 and GDSC2 were retained for visualization and analysis.

### 4.5 Laetitia's Adjuvant Chemotherapy‐Treated Colon Cancer Cohort, GSE39582

Microarray expression profiles for 585 colon cancer patients were downloaded from the GEO dataset using the *getGEO* function from the *GEOquery* package. Clinical information was extracted using the *pData* function. For genes associated with multiple probes, expression values were averaged. We identified 233 patients who received adjuvant chemotherapy for subsequent analysis. Prior to SVM classification, all expression values were *z*‐score normalized.

### 4.6 Anti‐BRAF‐Treated Melanoma Cohorts, GSE50509, GSE65185, GSE99898

Expression profiles of melanoma patients were retrieved from the GEO with accession numbers GSE50509, GSE65185, and GSE99898. Prognostic variables, including PFS, OS, and best response assessed according to the Response Evaluation Criteria in Solid Tumors (RECIST) version 1.1, were collected from the original publications and corresponding supplementary materials (OS data were unavailable for GSE65185). A total of 55 anti‐BRAF‐treated samples were selected for subsequent analysis. Expression matrices were *z*‐score normalized prior to SVM classification.

### 4.7 SVM Classifier

To reproduce the BAG classification in external CRC and melanoma cohorts, we developed an SVM classifier based on the BRAF25 signature. Following *z*‐score normalization, TCGA‐COAD and ‐SKCM expression matrices were partitioned into an 80% training set and a 20% test set using the *createDataPartition* function from the *caret* R package. Feature redundancy was assessed using the *findCorrelation* function (cutoff = 0.8), and no highly correlated genes were removed. We trained a Random Forest model on the training set to rank genes by importance scores. These ranked genes were then iteratively incorporated into a Radial Basis Function (RBF) SVM model using stepwise feature selection. Model tuning and performance optimization were conducted through fivefold cross‐validation using the *trainControl* function. The optimal SVM classifier was selected based on its accuracy, sensitivity, and specificity in classifying the test set.

### 4.8 BAGs Classification

The VST‐normalized BRAF25 expression matrix from TCGA was subjected to hierarchical clustering using the Ward.D2 agglomeration method. Resulting clusters were designated as BAG‐0 to BAG‐3, ordered by increasing mean BRAF25 expression value. To evaluate the discriminatory power of each founding BRAF signature, this clustering process was iteratively applied, and associations between BAG classifications and *BRAF* mutation status were assessed using chi‐squared tests. Somatic variants showing significant differences in frequency across BAG groups were identified using chi‐squared tests (*p*‐value < 0.05). Computing BRAF index for each sample was realized using the ssGSEA algorithm implemented in the *GSVA* R package. TCGA samples were dichotomized based on BRAF index to assess its discriminative power for *BRAF* mutation status. The AUROC value was calculated using the *pROC* package, and the convex hull generated using the*geometry* package was used to visualize the classifier boundaries. Given the class imbalance between *BRAF*‐mutant and WT samples in the CRC dataset, the AUPRC was calculated using the *PRROC* package. A radar plot summarizing the scaled AUROC and AUPRC values was generated using the *fmsb* package.

### 4.9 Survival Analyses

We initially employed univariate Cox proportional hazards models to evaluate the prognostic value of BAG labels. For multivariate analysis in Laetitia's colon cancer cohort, we constructed full models incorporating established clinical covariates, including age, gender, TNM stage, tumor location, and mismatch repair (MMR) status, alongside BAG labels or *BRAF* mutation status. For the TCGA‐SKCM cohort, we integrated Bagaev's Molecular Functional Portrait (MFP) classification system [49], which characterizes distinct TME subtypes: immune‐enriched, fibrotic (IE/F), immune‐enriched, non‐fibrotic (IE), fibrotic (F), and immune‐depleted (D). To account for potential non‐linear effects of age, this variable was modeled using restricted cubic splines with three knots. To explore the additional predictive effects of BAG labels beyond *BRAF* mutations, we generated an interaction term (BAG × BRAF.MUT) and included it in the multivariate models. All Cox models were fitted using the *coxph* function from the *survival* R package. For TCGA pan‐cancer analyses, we triple‐split samples according to the BRAF index and assessed associations with both OS and PFS.  Survival curves across these stratified groups were compared using the log‐rank test via the *survdiff* function and visualized with the *ggsurvplot* function from the *survminer* R package.

### 4.10 Pan‐Cancer BRAF25 Analysis

To enable cross‐cancer comparison of BRAF25 expression, we applied sample‐wise *z*‐score normalization to VST‐transformed expression matrices from 32 TCGA cohorts before data integration. The association between BRAF pathway mutational burden and BRAF activity was evaluated by calculating the cohort‐level average mutation frequency (percentage of samples with BRAF pathway mutations) and correlating it with the mean BRAF index using Spearman's rank correlation. Samples were categorized as BRAF pathway‐mutated if they carried mutations in any gene involved in the BRAF signaling pathway. To examine discordance between BRAF activity and canonical *BRAF* mutations, BRAF index values were compared between samples harboring *EGFR*, *ERBB2*, or *KRAS* mutations (as alternative BRAF pathway activators) and WT controls. The collective impact of *RAF* family mutations was examined by combining variant calls for *ARAF*, *BRAF*, and *RAF1* and comparing BRAF index values between mutant and WT groups.

### 4.11 Statistical Analysis

Group comparisons for non‐normally distributed data were performed using the Wilcoxon rank‐sum test. Differences were considered statistically significant at *p* < 0.05. The symbols used to denote significance are as follows: *p < 0.05, **p < 0.01, ***p < 0.001, and n.s. (not significant). Detailed protocols for cell culture and additional methodologies are provided in the Supporting Information.

## Author Contributions

Conceptualization: K.D.Y., Y.Y., and X.W.B. Methodology: K.D.Y. and Y.Y. Software: K.D.Y. and J.B.L. Validation: Y.Y. Formal analysis: S.H.F., L.J.D., and K.D.Y. Investigation: L.J.D., S.H.F., and K.D.Y. Resources: X.W.B. Data curation: J.H.Y. and F.L. Writing – original draft preparation: K.D.Y. Writing – review and editing: X.W.B. Visualization: Y.Y. Supervision: X.W.B. Project administration: K.D.Y. and X.W.B. All authors confirmed the underlying data and reviewed and approved the submission of the manuscript.

## Funding

This research was supported by the National Natural Science Foundation of China (Nos. 82303551 and 82403754), the Natural Science Foundation of Chongqing (No. CSTB2024NSCQ‐MSX0499), and the Natural Science Foundation of Hainan Province (No. 825QN580).

## Ethics Statement

Ethical approval for this study was waived because it exclusively involved the analysis of de‐identified data from public repositories (e.g., TCGA and GEO) in accordance with their specific data use policies.

## Conflicts of Interest

The authors declare no conflicts of interest.

## Supporting information




**Supporting Figure 1**: (A) Heatmap visualizing the gene overlaps among different BRAF founder signatures. Each row represents a gene, and each column corresponds to a distinct signature. Dark blue blocks indicate the presence of a gene in a given signature. (B) Scatter plots demonstrating the filtering process for each BRAF founder signature based on normalized CCLE expression data. The log coefficient of variation (y‐axis) is plotted against the mean gene expression (x‐axis). A loess curve (black line) is fitted to the data. Genes with a log coefficient of variation above the fitted line and mean gene expression >6 are marked in red. Discarded genes are labeled in black, and non‐signature genes are shown in grey. (C) Heatmap of normalized expression profiles from 216 CCLE cell lines (rows) stratified by each gene signature (columns). The three clusters are labeled as BRAF‐high, ‐unclassified (medium), and ‐low according to the mean signature expression. *BRAF* mutational status is indicated in rose red on the right. (D) Volcano plots showing the significance and magnitude of differential expression between BRAF‐high and BRAF‐low groups across various signatures, including shBRAF, TCGA‐BRAFV600E, Melanoma BRAF pathway, Colon BRAF pathway, and Vemurafenib signatures. DEGs with a ‐log10 (false discovery rate) FDR>10 and a positive log2 fold‐change>1 (limma analysis) are labeled in magenta.
**Supporting Figure 2**: (A‐D) Immunoblot analysis of BRAFV600E expression in colorectal cancer (A), glioma (B), thyroid cancer (C) and melanoma cell lines (D). (E) Heatmap of normalized expression profiles for colorectal cancer cell lines (columns), stratified by the BRAF25 signature genes (rows). Cell lines are annotated by BRAF mutation status using distinct colors.
**Supporting Figure 3**: (A‐B) Heatmaps showing the mean expression levels of BRAF25 genes (BRAF activity) alongside cell‐type‐specific lineage markers across various cell subsets, as identified from single‐cell RNA sequencing datasets of human tumors: melanoma (A, GSE72056), lung adenocarcinoma (B, GSE131907), colorectal cancer (C, GSE200997) and papillary thyroid cancer (D, GSE184362). CAF, Cancer‐associated fibroblast; NK, Natural killer cell.
**Supporting Figure 4**: (A) Violin plot showing the distribution of BRAF index values in BRAF wild‐type and mutant tumors. Embedded boxplots indicate the median (horizontal line) and interquartile range (shaded box). (B) Heatmap illustrating normalized CPTAC proteomic data from COAD, stratified by the BRAF25 signature (column). (C‐E) Heatmaps illustrating VST‐normalized CPTAC or OncoSG RNA‐seq data from COAD (C), glioblastoma (GBM) (D) and lung adenocarcinoma (E) samples, stratified by the BRAF25 signature (column). (F‐H) Receiver operating characteristic (ROC) curves evaluating the classification performance of BRAF founder signatures and the BRAF25 signature (marked with an asterisk) across the COAD (F), THCA (G), and SKCM (H) datasets. Each colored line represents a distinct signature, with sensitivity on the y‐axis and 1‐specificity (false positive rate) on the x‐axis. (I) Precision‐recall curve (PRC) illustrating the classification performance of BRAF founder signatures and the BRAF25 signature (marked with an asterisk) in the COAD dataset. Each colored line represents a distinct signature, with precision on the y‐axis and recall on the x‐axis. (J) Cumulative frequency distribution of specific *BRAF* mutational variants resulting in amino acid substitutions across BAGs. (K) Violin plots showing the distributions of phosphorylated ERK1/2 (MAPK‐pT202Y204) MEK1 (pS217S221) and EGFR (pY1068) levels across the BAGs. Each embedded boxplot indicates the median (horizontal bar) and interquartile range (shaded box).
**Supporting Figure 5**: (A) Forest plot showing the results of a multivariate Cox proportional hazards analysis for OS across BAG classification in the TCGA‐SKCM cohort. Classification is based on the BRAF25 signature, with BAG‐0 serving as the reference group. (B) Kaplan‐Meier plot comparing OS between BAG‐3 patients (defined by the BRAF25 signature) and BAG‐3 patients unmatched with C3 (defined by the melanoma BRAF pathway signature) in the TCGA‐SKCM cohort. (C) Frequency histogram showing the distribution of BRAF mutation status across predicted BAGs in the chemotherapy‐treated Laetitia's colon cancer cohort. (D) Kaplan‐Meier plots comparing OS between BAG‐3 and BAG‐0 to BAG‐2 patients in the chemotherapy‐treated Laetitia's colon cancer cohort. (E) Forest plot of multivariate Cox proportional hazards analysis for OS across BAG classifications in the chemotherapy‐treated colon cancer cohort, with BAG‐0 serving as the reference group.
**Supporting Figure 6**: (A) Density plot depicting the density distribution of pairwise Pearson correlation coefficients among BRAF25 genes in the TCGA‐COAD cohort. (B) Heatmap showing pairwise Pearson correlation coefficients for all BRAF25 genes in the TCGA‐COAD cohort, with color gradients representing the strength of correlation. (C) SVM classifier accuracy (y‐axis) plotted against the rank number of BRAF25 genes (x‐axis) used in the classifier construction. Gene ranking is based on importance scores derived from a Random Forest (RF) model trained on the TCGA‐COAD cohort data. Classifier accuracy was evaluated on a 20% hold‐out test set. The gene‐panel size with the highest accuracy value is denoted. (D) SVM classifier sensitivity (left panel) and specificity (right panel) are plotted against the number of BRAF25 genes (x‐axis), following the same ranking strategy as in Figure 7C.
**Supporting Figure 7**: (A) Volcano plot showing the significance (y‐axis) and magnitude of difference (x‐axis) in IC50 values for compounds in the GDSC drug library between BRAF mutant and wild‐type cell lines, based on CCLE cell line data. Compounds exhibiting significantly increased sensitivity in BRAF‐mutant lines (log2 fold‐change >1 and FDR < 0.05 after Benjamini‐Hochberg correction) are highlighted in red. (B) Venn diagram showing the overlay of sensitive drug candidates associated with the ERK‐MAPK signaling term from the three comparisons: BRAF mutant versus wild‐type cell lines (blue), BRAF‐pathway mutated versus wild‐type cell lines (yellow), and BRAF25‐high versus BRAF25‐low expression cell lines (green), in the context of CCLE cell lines data.
**Supporting Figure 8**: (A‐D) Heatmaps showing the normalized BRAF25 gene expression across four cancer types, including glioma (A), melanoma (B) thyroid cancer (C), and colorectal cancer (D). For each cancer type, both BRAFV600E OE and anti‐BRAF inhibitor (BRAFi) treatment groups are compared to their respective controls. Each subgroup includes three independent biological replicates.
**Supporting Figure 9**: (A‐B) Cell viability of three BRAF wild‐type (A) and mutant (B) colorectal cancer cell lines following 24‐hour treatment with BCI. (C) Bar plot showing the IC50 values of BRAF wild‐type and mutant colorectal cancer cell lines after BCI exposure.
**Supporting Figure 10**: (A) Density plot illustrating the distribution of *BRAF* mutation frequencies across TCGA cancer cohorts. The six top cohorts with the highest mutation frequencies are labeled. (B) Violin plots showing the distribution of BRAF index values in the TCGA‐LGG, ‐LUAD, and ‐LUSC cohorts, stratified by BRAF mutation status. Boxes within represent the median and interquartile range (IQR), with whiskers extending to ±1.5×IQR, and outliers are individually labeled. (C) Violin plots illustrating the distribution of BRAF index values in the TCGA‐COAD, ‐LUAD, ‐LUSC, ‐SKCM, and ‐THCA cohorts, categorized by RAF isoform mutation status. Boxes within represent the median and interquartile range (IQR), with whiskers extending to ±1.5×IQR, and outliers are individually labeled. ***, *p*<0.001; **, *p*<0.01; n.s., not significant.

**Supporting Table 1**: BRAF founder signature information including gene number, species of origin, organ, references, and methods of establishment.

**Supporting Table 2**: mco270591‐sup‐0003‐TableS2.xlsx.

**Supporting Table 3**: mco270591‐sup‐0004‐TableS3.xlsx.

**Supporting Table 4**: AUPRC and AUROC values for the nine BRAF signatures.

**Supporting Table 5**: mco270591‐sup‐0006‐TableS5.xlsx.

## Data Availability

All the data that support the findings of this study are available from the corresponding author upon reasonable request.
