# Supplementary material for: Unveiling a BRAF Signature Proficient in Accurately Capturing Oncogenic Activity and Guiding Prognostic Prediction Across Multiple Cancers
Source: MedComm (2020). 2026 Feb 3;7(2):e70591. doi: 10.1002/mco2.70591 (PMC12868921; doi:10.1002/mco2.70591)
Supplement: Supplementary file 1 — Supporting Figure 1: (A) Heatmap visualizing the gene overlaps among different BRAF founder signatures. Each row represents a gene, and each column corresponds to a distinct signature. Dark blue blocks indicate the presence of a gene in a given signature. (B) Scatter plots demonstrating the filtering process for each BRAF founder signature based on normalized CCLE expression data. The log coefficient of variation (y‐axis) is plotted against the mean gene expression (x‐axis). A loess curve (black line) is fitted to the data. Genes with a log coefficient of variation above the fitted line and mean gene expression >6 are marked in red. Discarded genes are labeled in black, and non‐signature genes are shown in grey. (C) Heatmap of normalized expression profiles from 216 CCLE cell lines (rows) stratified by each gene signature (columns). The three clusters are labeled as BRAF‐high, ‐unclassified (medium), and ‐low according to the mean signature expression. BRAF mutational status is indicated in rose red on the right. (D) Volcano plots showing the significance and magnitude of differential expression between BRAF‐high and BRAF‐low groups across various signatures, including shBRAF, TCGA‐BRAFV600E, Melanoma BRAF pathway, Colon BRAF pathway, and Vemurafenib signatures. DEGs with a ‐log10 (false discovery rate) FDR>10 and a positive log2 fold‐change>1 (limma analysis) are labeled in magenta. Supporting Figure 2: (A‐D) Immunoblot analysis of BRAFV600E expression in colorectal cancer (A), glioma (B), thyroid cancer (C) and melanoma cell lines (D). (E) Heatmap of normalized expression profiles for colorectal cancer cell lines (columns), stratified by the BRAF25 signature genes (rows). Cell lines are annotated by BRAF mutation status using distinct colors. Supporting Figure 3: (A‐B) Heatmaps showing the mean expression levels of BRAF25 genes (BRAF activity) alongside cell‐type‐specific lineage markers across various cell subsets, as identified from single‐cell RNA sequencing da [file MCO2-7-e70591-s003.docx]

*Original Article*

Unveiling a BRAF Signature Proficient in Accurately Capturing Oncogenic Activity and Guiding Prognostic Prediction across Multiple Cancers

Kaidi Yang^1,2,*^, Shihui Fu^3^, Jingbing Liang^4^, Lijuan Ding^1^, Junhao You^1^, Fang Li^1^, Ye Yuan^5,*^, Xiu-wu Bian^6,*^

**Author affiliations**

^1^Department of Oncology, Hainan Hospital of Chinese People’s Liberation Army General Hospital, Sanya, People's Republic of China

^2^ Institute of Pathology and Southwest Cancer Center, Southwest Hospital, and The Key Laboratory of Tumor Immunopathology (Third Military Medical University), The Ministry of Education of China, Chongqing, People's Republic of China

^3^Department of Cardiology, Hainan Hospital of Chinese People’s Liberation Army General Hospital, Sanya, People's Republic of China

^4^Department of Health Management Center, Hainan Hospital of Chinese People’s Liberation Army General Hospital, Sanya, People's Republic of China

^5^Department of Medical Oncology, Chongqing University Cancer Hospital, Chongqing, People's Republic of China

^6^Institute of Pathology and Southwest Cancer Center, Southwest Hospital, Third Military Medical University (Army Medical University), and The Key Laboratory of Tumor Immunopathology, The Ministry of Education of China, Chongqing, People's Republic of China

***Correspondence:** Kaidi Yang (lampirl@163.com) | Yuan Ye (yuanye02@foxmail.com) | Xiu-Wu Bian (bianxiuwu@263.net)

**Running title:** Generation of BRAF signature capable of capturing oncogenic activity.


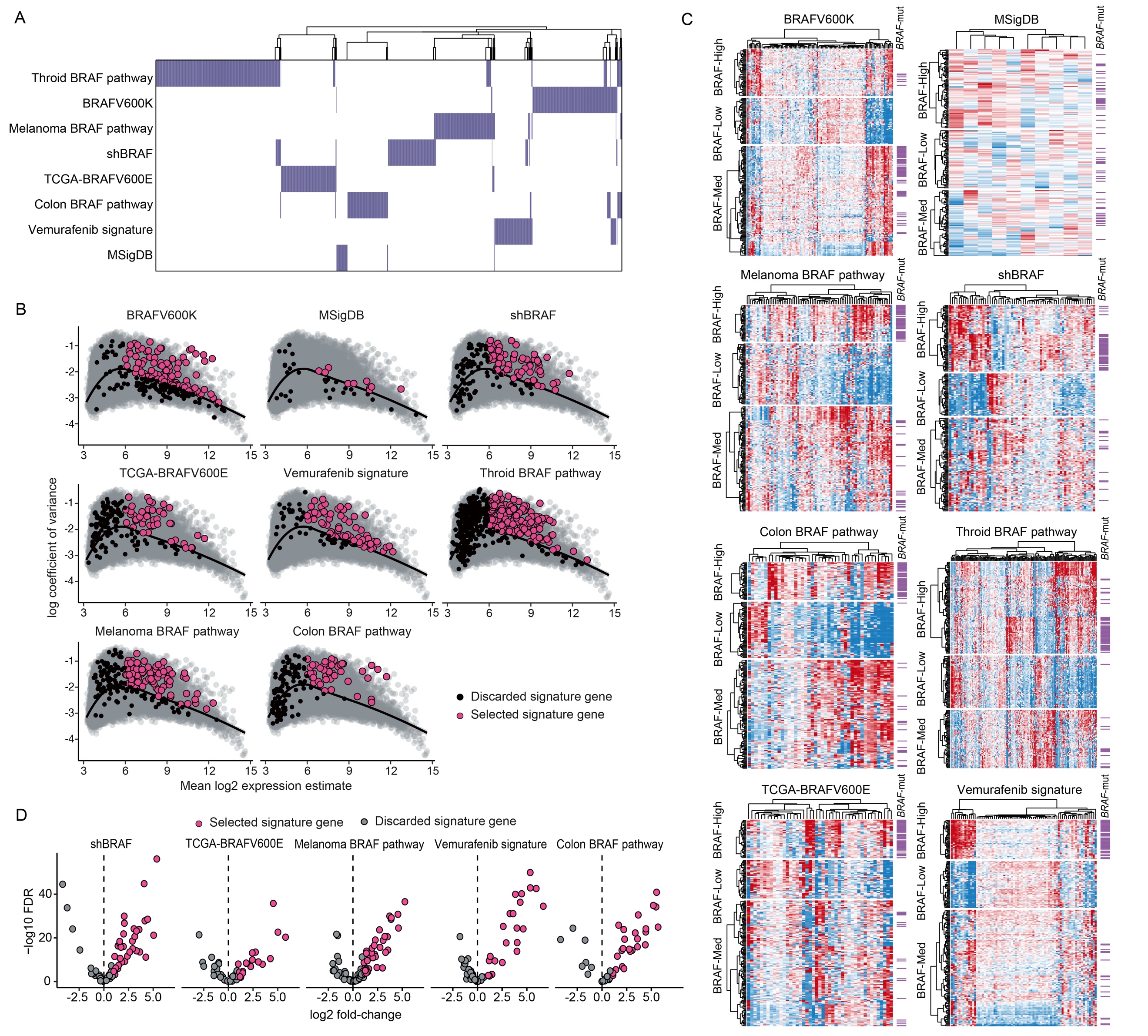


**Figure S1** (A) Heatmap visualizing the gene overlaps among different BRAF founder signatures. Each row represents a gene, and each column corresponds to a distinct signature. Dark blue blocks indicate the presence of a gene in a given signature. (B) Scatter plots demonstrating the filtering process for each BRAF founder signature based on normalized CCLE expression data. The log coefficient of variation (y-axis) is plotted against the mean gene expression (x-axis). A loess curve (black line) is fitted to the data. Genes with a log coefficient of variation above the fitted line and mean gene expression >6 are marked in red. Discarded genes are labeled in black, and non-signature genes are shown in grey. (C) Heatmap of normalized expression profiles from 216 CCLE cell lines (rows) stratified by each gene signature (columns). The three clusters are labeled as BRAF-high, -unclassified (medium), and -low according to the mean signature expression. *BRAF* mutational status is indicated in rose red on the right. (D) Volcano plots showing the significance and magnitude of differential expression between BRAF-high and BRAF-low groups across various signatures, including shBRAF, TCGA-BRAFV600E, Melanoma BRAF pathway, Colon BRAF pathway, and Vemurafenib signatures. DEGs with a -log10 (false discovery rate) FDR＞10 and a positive log2 fold-change＞1 (limma analysis) are labeled in magenta.


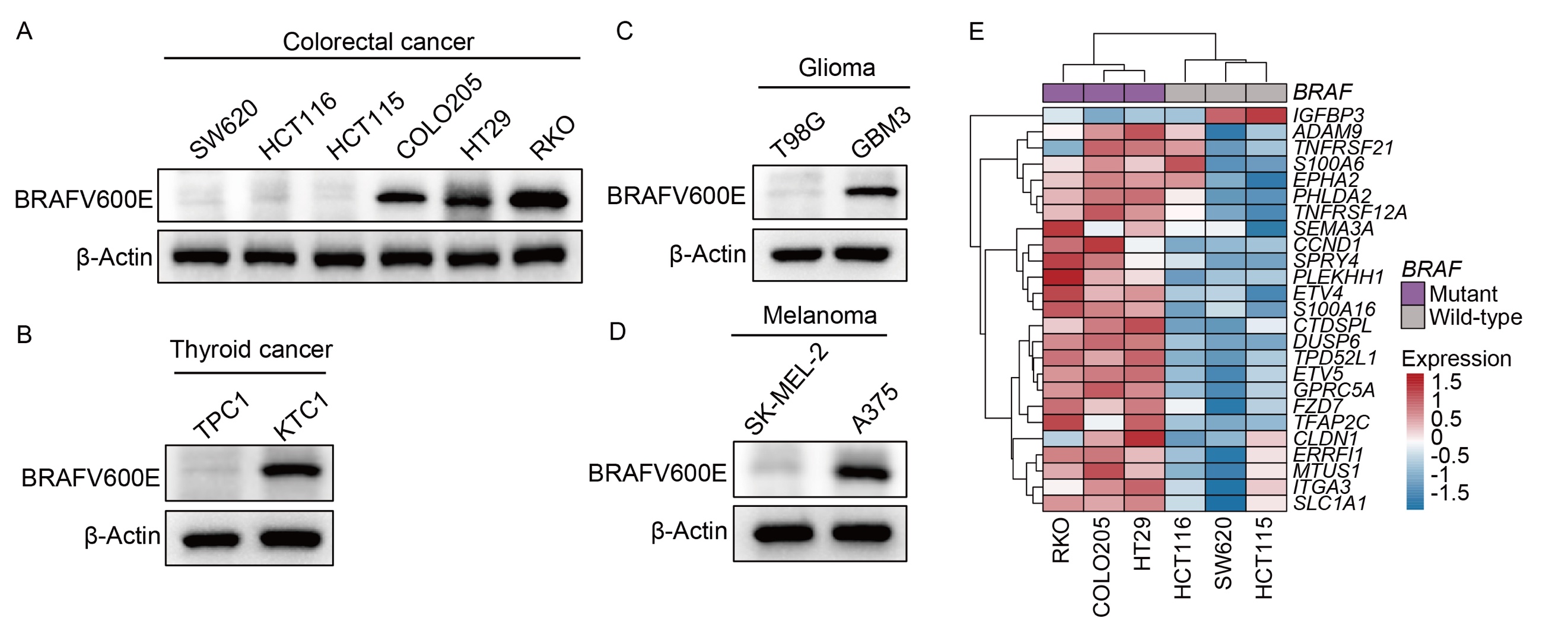


**Figure S2** (A-D) Immunoblot analysis of BRAFV600E expression in colorectal cancer (A), glioma (B), thyroid cancer (C) and melanoma cell lines (D). (E) Heatmap of normalized expression profiles for colorectal cancer cell lines (columns), stratified by the BRAF25 signature genes (rows). Cell lines are annotated by *BRAF* mutation status using distinct colors.

**
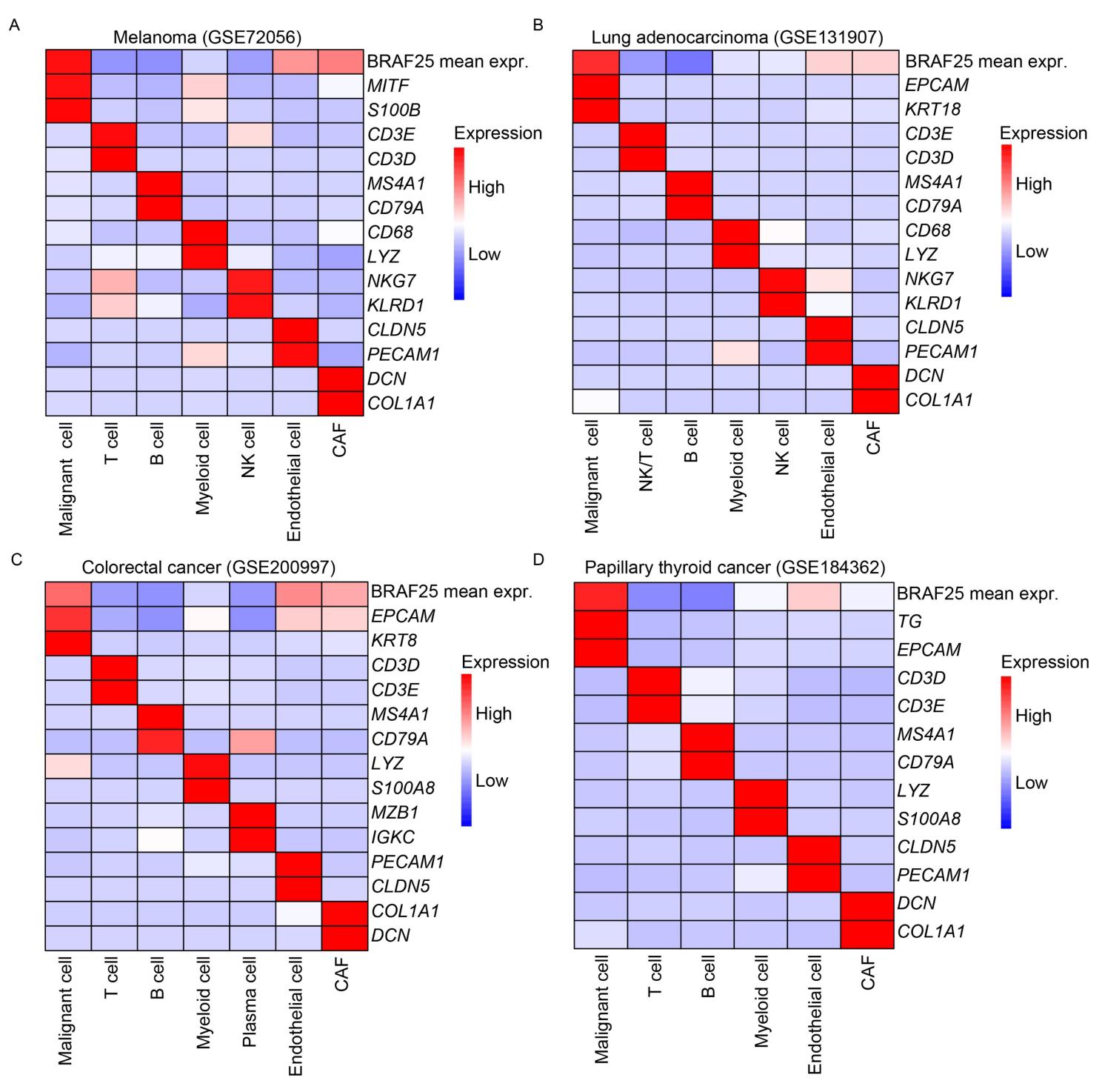
 Figure S3** (A-B) Heatmaps showing the mean expression levels of BRAF25 genes (BRAF activity) alongside cell-type-specific lineage markers across various cell subsets, as identified from single-cell RNA sequencing datasets of human tumors: melanoma (A, GSE72056), lung adenocarcinoma (B, GSE131907), colorectal cancer (C, GSE200997) and papillary thyroid cancer (D, GSE184362). CAF, Cancer-associated fibroblast; NK, Natural killer cell.


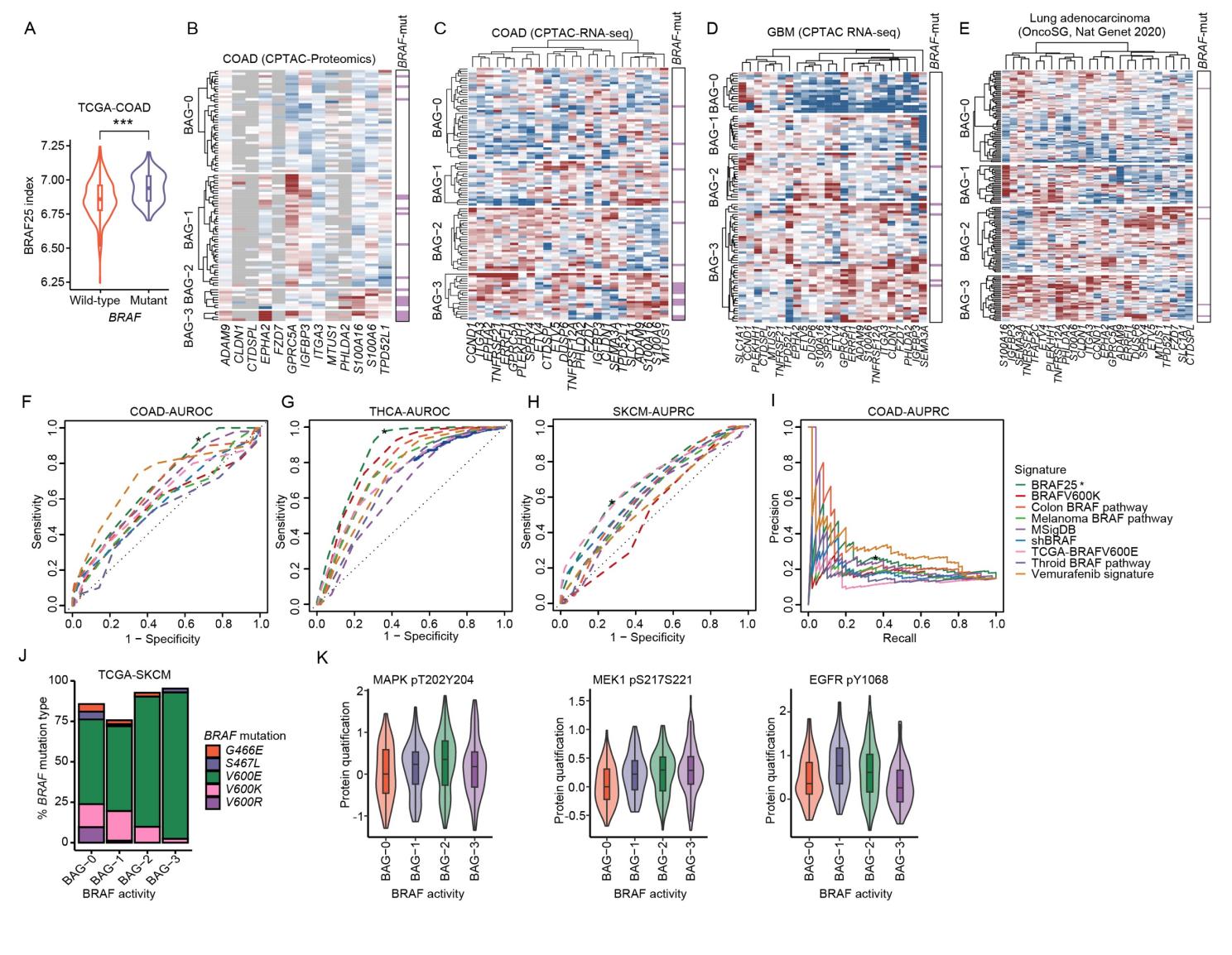


**Figure S4** (A) Violin plot showing the distribution of BRAF index values in *BRAF* wild-type and mutant tumors. Embedded boxplots indicate the median (horizontal line) and interquartile range (shaded box). (B) Heatmap illustrating normalized CPTAC proteomic data from COAD, stratified by the BRAF25 signature (column). (C-E) Heatmaps illustrating VST-normalized CPTAC or OncoSG RNA-seq data from COAD (C), glioblastoma (GBM) (D) and lung adenocarcinoma (E) samples, stratified by the BRAF25 signature (column). (F-H) Receiver operating characteristic (ROC) curves evaluating the classification performance of BRAF founder signatures and the BRAF25 signature (marked with an asterisk) across the COAD (F), THCA (G), and SKCM (H) datasets. Each colored line represents a distinct signature, with sensitivity on the y-axis and 1-specificity (false positive rate) on the x-axis. (I) Precision-recall curve (PRC) illustrating the classification performance of BRAF founder signatures and the BRAF25 signature (marked with an asterisk) in the COAD dataset. Each colored line represents a distinct signature, with precision on the y-axis and recall on the x-axis. (J) Cumulative frequency distribution of specific *BRAF* mutational variants resulting in amino acid substitutions across BAGs. (K) Violin plots showing the distributions of phosphorylated ERK1/2 (MAPK-pT202Y204) MEK1 (pS217S221) and EGFR (pY1068) levels across the BAGs. Each embedded boxplot indicates the median (horizontal bar) and interquartile range (shaded box).


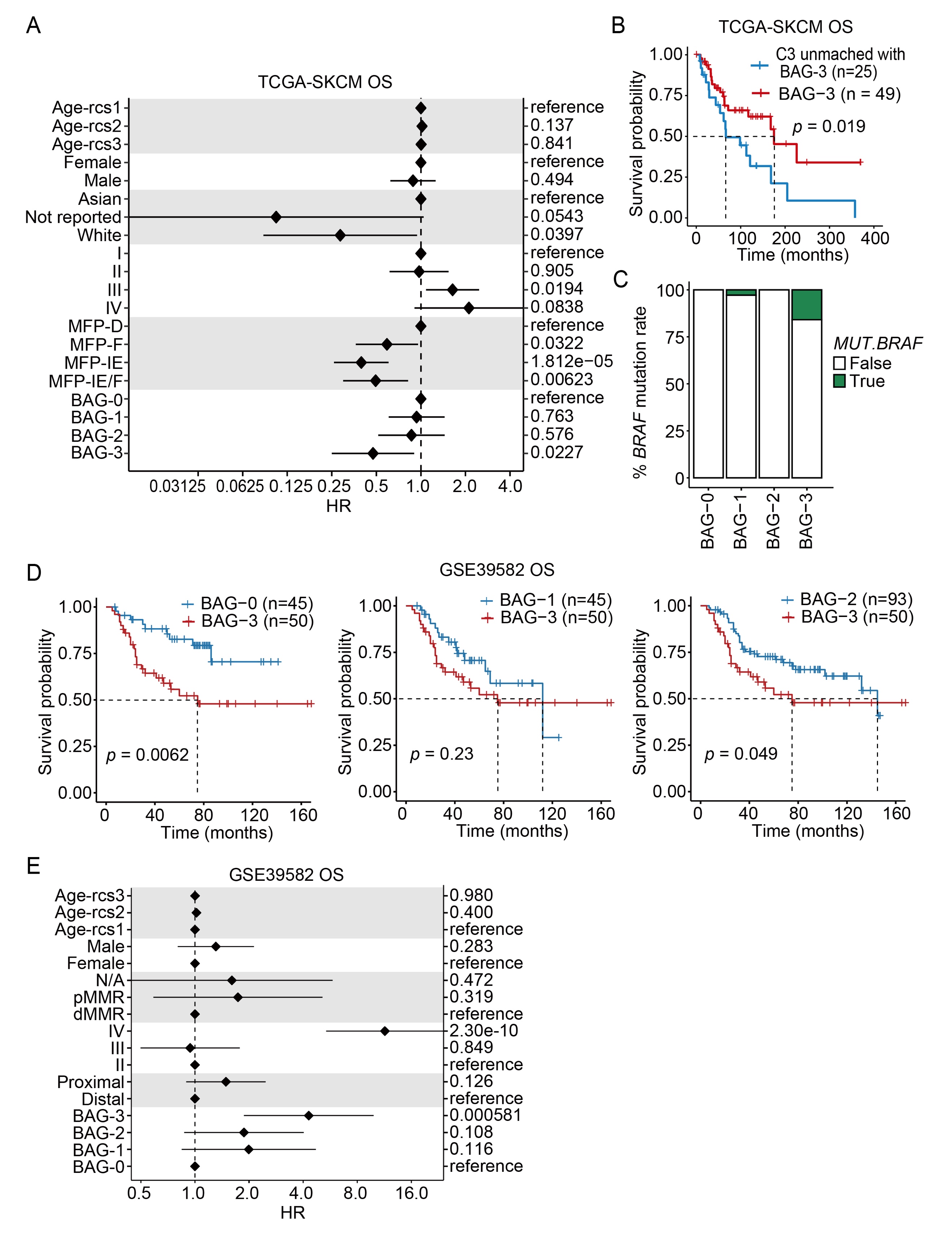


**Figure S5** (A) Forest plot showing the results of a multivariate Cox proportional hazards analysis for OS across BAG classification in the TCGA-SKCM cohort. Classification is based on the BRAF25 signature, with BAG-0 serving as the reference group. (B) Kaplan-Meier plot comparing OS between BAG-3 patients (defined by the BRAF25 signature) and BAG-3 patients unmatched with C3 (defined by the melanoma BRAF pathway signature) in the TCGA-SKCM cohort. (C) Frequency histogram showing the distribution of *BRAF* mutation status across predicted BAGs in the chemotherapy-treated Laetitia’s colon cancer cohort. (D) Kaplan-Meier plots comparing OS between BAG-3 and BAG-0 to BAG-2 patients in the chemotherapy-treated Laetitia’s colon cancer cohort. (E) Forest plot of multivariate Cox proportional hazards analysis for OS across BAG classifications in the chemotherapy-treated colon cancer cohort, with BAG-0 serving as the reference group.


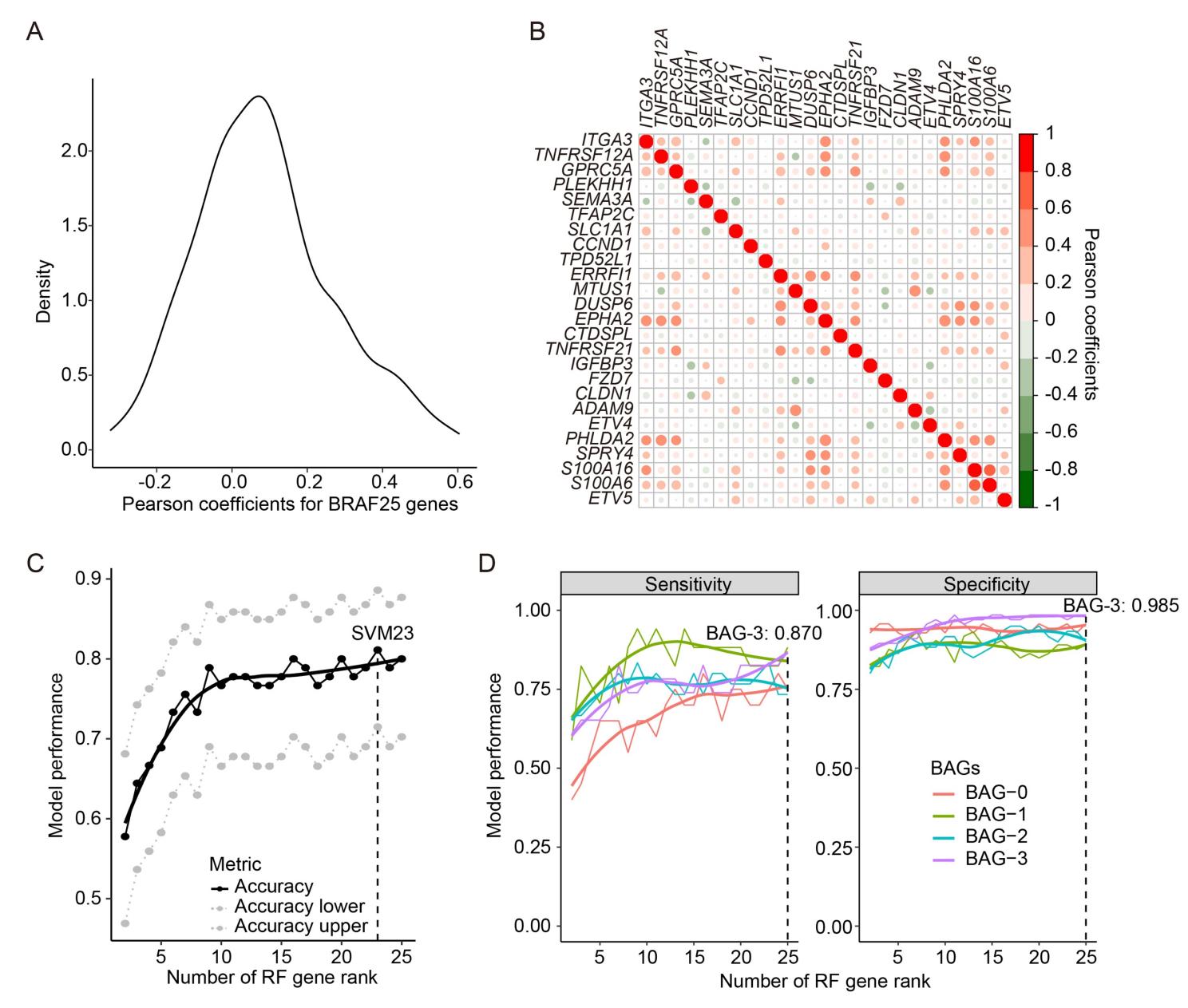


**Figure S6** (A) Density plot depicting the density distribution of pairwise Pearson correlation coefficients among BRAF25 genes in the TCGA-COAD cohort. (B) Heatmap showing pairwise Pearson correlation coefficients for all BRAF25 genes in the TCGA-COAD cohort, with color gradients representing the strength of correlation. (C) SVM classifier accuracy (y-axis) plotted against the rank number of BRAF25 genes (x-axis) used in the classifier construction. Gene ranking is based on importance scores derived from a Random Forest (RF) model trained on the TCGA-COAD cohort data. Classifier accuracy was evaluated on a 20% hold-out test set. The gene-panel size with the highest accuracy value is denoted. (D) SVM classifier sensitivity (left panel) and specificity (right panel) are plotted against the number of BRAF25 genes (x-axis), following the same ranking strategy as in Figure 7C.


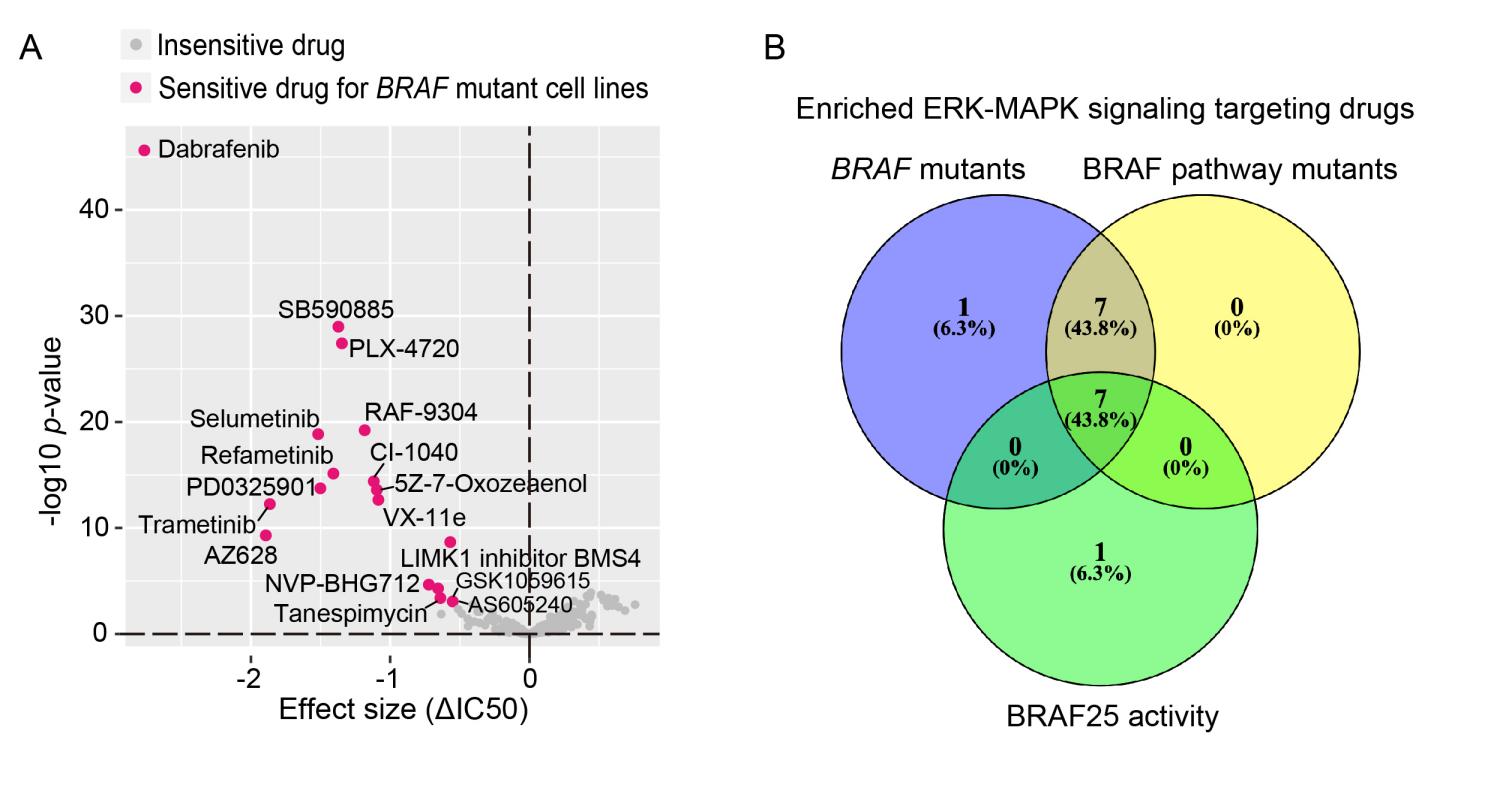


**Figure S7** (A) Volcano plot showing the significance (y-axis) and magnitude of difference (x-axis) in IC50 values for compounds in the GDSC drug library between *BRAF* mutant and wild-type cell lines, based on CCLE cell line data. Compounds exhibiting significantly increased sensitivity in BRAF-mutant lines (log2 fold-change >1 and FDR < 0.05 after Benjamini-Hochberg correction) are highlighted in red. (B) Venn diagram showing the overlay of sensitive drug candidates associated with the ERK-MAPK signaling term from the three comparisons: *BRAF* mutant versus wild-type cell lines (blue), BRAF-pathway mutated versus wild-type cell lines (yellow), and BRAF25-high versus BRAF25-low expression cell lines (green), in the context of CCLE cell lines data.


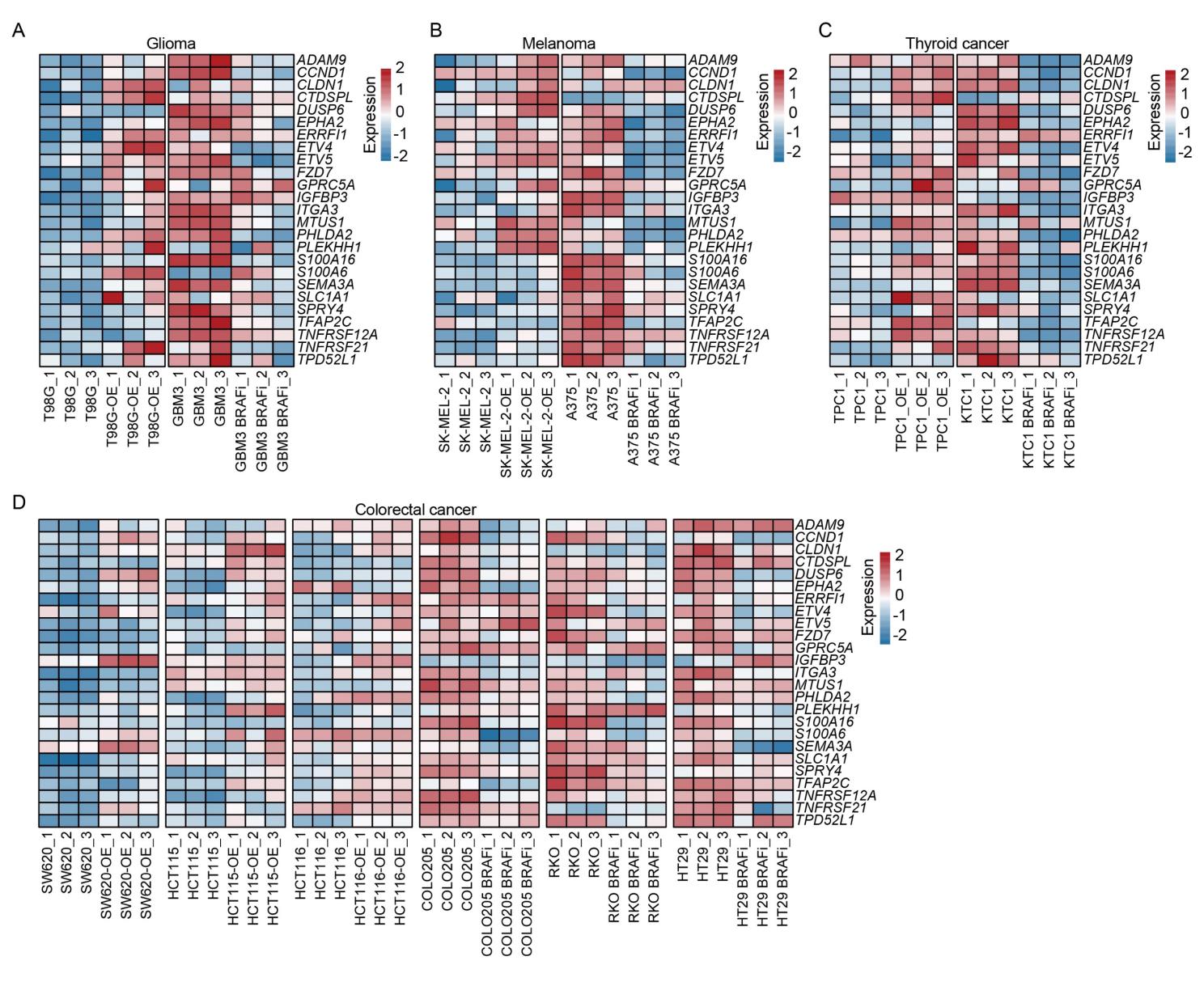


**Figure S8** (A-D) Heatmaps showing the normalized BRAF25 gene expression across four cancer types, including glioma (A), melanoma (B) thyroid cancer (C), and colorectal cancer (D). For each cancer type, both *BRAFV600E* OE and anti-BRAF inhibitor (BRAFi) treatment groups are compared to their respective controls. Each subgroup includes three independent biological replicates.

**
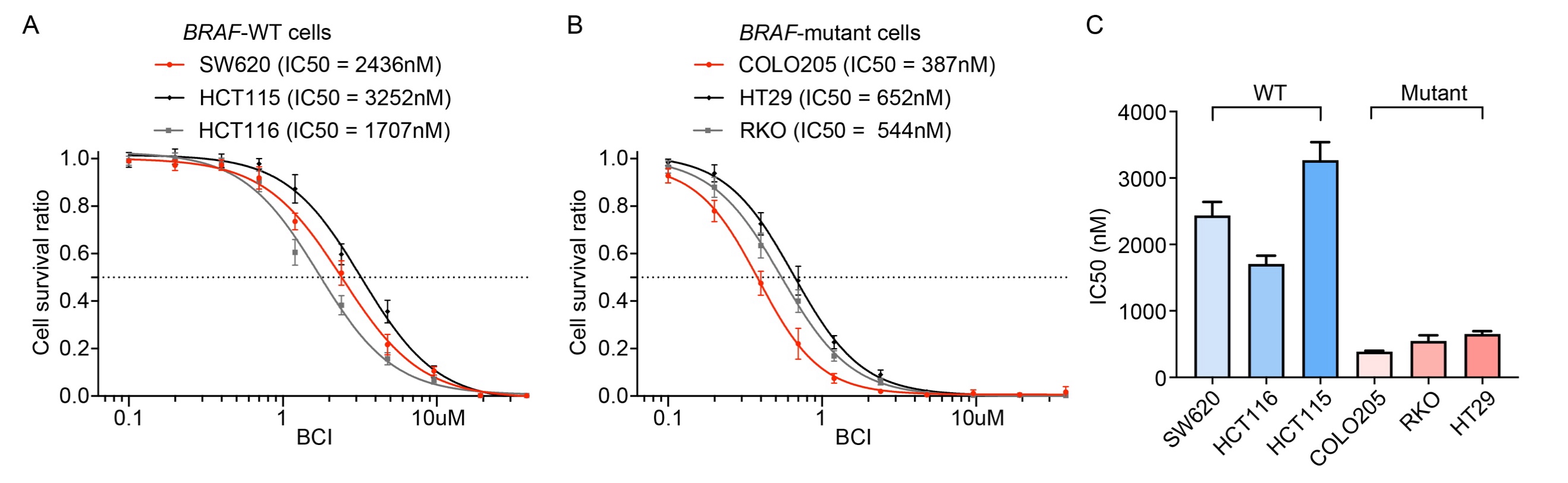
**

**Figure S9** (A-B) Cell viability of three *BRAF* wild-type (A) and mutant (B) colorectal cancer cell lines following 24-hour treatment with BCI. (C) Bar plot showing the IC50 values of *BRAF* wild-type and mutant colorectal cancer cell lines after BCI exposure.


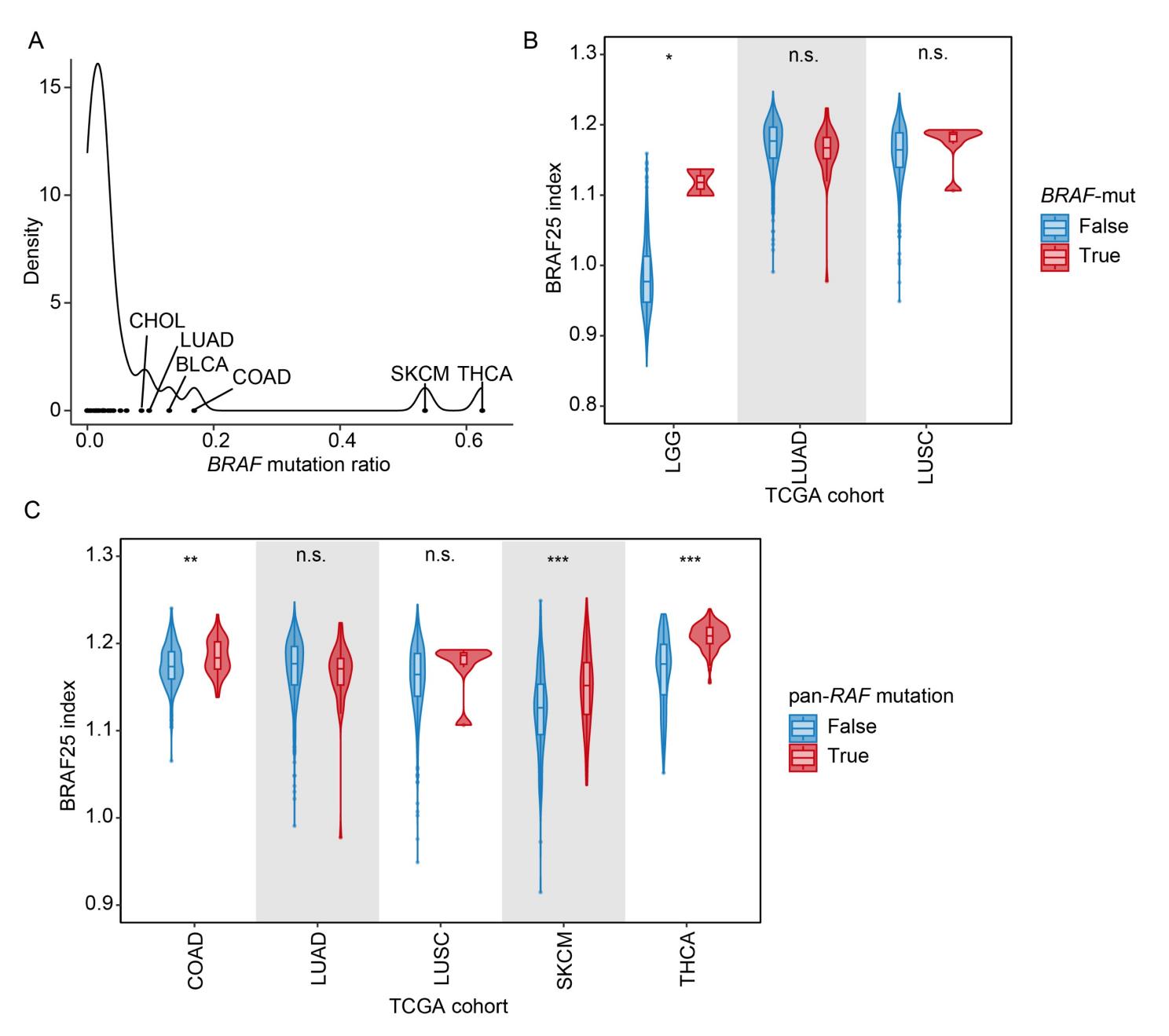


**Figure S10** (A) Density plot illustrating the distribution of *BRAF* mutation frequencies across TCGA cancer cohorts. The six top cohorts with the highest mutation frequencies are labeled. (B) Violin plots showing the distribution of BRAF index values in the TCGA-LGG, -LUAD, and -LUSC cohorts, stratified by *BRAF* mutation status. Boxes within represent the median and interquartile range (IQR), with whiskers extending to ±1.5×IQR, and outliers are individually labeled. (C) Violin plots illustrating the distribution of BRAF index values in the TCGA-COAD, -LUAD, -LUSC, -SKCM, and -THCA cohorts, categorized by *RAF* isoform mutation status. Boxes within represent the median and interquartile range (IQR), with whiskers extending to ±1.5×IQR, and outliers are individually labeled. ***, *p*＜0.001; **, *p*＜0.01; n.s., not significant.

**Materials and methods**

*Single-cell analyses for identifying tumor-dominant genes*

To minimize confounding signals from immune and stroma cells, we sought to determine the neoplastic-specific expression of the 134 BRAF meta-signature through scRNA-seq analyses. Four scRNA-seq tumor datasets corresponding to the analyzed cell line types (lung, skin, colon, and thyroid) were obtained from the Gene Expression Omnibus (GEO) with the accession numbers GSE131907, GSE72056, GSE200997, and GSE184362. Raw expression matrices were retrieved and transformed into *SingleCellExperiment* objects using the *Seurat* R pack­age. After filtering out low-quality cells and performing data normalization, high-variance genes were selected for linear dimensionality reduction. The optimal number of dimensions of principal components was determined using an elbow plot and subsequently used for constructing a nearest-neighbor graph via the *FindNeighbors* function*.* To retain resolution sufficient for identifying key cellular compartments, including epithelial (neoplastic) cells, NK/T cells, B cells, tumor-associated macrophages (TAMs), and fibroblasts, we applied a coarse clustering strategy that preserved approximately 6-8 distinct clusters.

Following the cell-type annotation, we identified DEGs in the epithelial subset against other subsets using the “*FindMarkers*” function (min.pct = 0.1, log2FC ≥ 1). The 134 genes from the BRAF meta-signature were ranked by adjusted *p*-values derived from scRNA-seq analyses, resulting in four ranked gene lists.

*TCGA pan-cancer atlas*

RNA-Seq STAR count data for all TCGA cohorts were obtained from the TCGA legacy repository using the *TCGAbiolinks* package and normalized using the *varianceStabilizingTransformation* function from the *DESeq2* package. Only samples annotated as primary solid tumors were included. For melanoma, only metastatic tumor samples were retained. Formalin-fixed paraffin-embedded (FFPE) samples (annotated as “01B”) were excluded due to low sequencing quality, as recommended by the TCGA official guidelines. Somatic mutation data were curated from the study by Sanchez-Vega et al ^[1]^. *BRAF* mutations encoding specific protein variants were retrieved from the *cBioPortal* database (<https://www.cbioportal.org/>).

*TCGA proteomics data*

Level 4 normalized reverse-phase protein array (RPPA) data for COAD patients were obtained via the *TCGAbiolinks* R package. Patients were stratified into BRAF25-based BAG subtypes according to predefined classifications. Phosphoprotein levels of MEK1 (p-MEK1, S217/S221), phosphorylated MAPK (p-MAPK, T202/Y204), and phosphorylated EGFR (p-EGFR, Y1068) were compared across BAG groups, with BAG-0 serving as the control.

*Cell culture*

The human glioblastoma (GBM) cell line T98G (ATCC-CRL-1690) and the primary GBM cell line GBM3 were authenticated as previously described ^[2]^. Human thyroid cancer cell lines (TPC1, KTC1), melanoma cell lines (A375, SK-MEL-2), and colorectal carcinoma cell lines (COLO205, SW620, HCT115, HCT116, RKO, and HT29) were purchased from Procell (Wuhan, China). All cell lines used in this study were authenticated by short tandem repeat (STR) analysis to confirm their identity and were routinely tested to ensure they were mycoplasma-free. All cells were cultured in Dulbecco’s Modified Eagle’s Medium (DMEM, Cat. No. 10566016, Gibco, Grand Island, NY, USA) supplemented with 10% heat-inactivated fetal bovine serum (Cat. No.16000-044, Gibco), 100 U/mL penicillin, and 100 μg/mL streptomycin at 37 °C in a humidified incubator with 5% CO₂. For BRAFV600E inhibitor treatment, cancer cells were exposed to Vemurafenib (10 μM, Cat. No. S1267, Selleck Chemicals; Houston, TX, USA) for 24 h.

*Immunoblot*

Cultured cells were lysed on ice using RIPA lysis buffer supplemented with protease inhibitor cocktails (Cat. No. MA0151, Meilunbio, China; Cat. No. MB2678, Meilunbio, China, dilution 1:100). Equal amounts of protein were resolved on a 10% SDS-PAGE gel (Cat. No. PG112, EpiZyme, China) alongside a molecular weight marker (Cat. No. WJ102, EpiZyme, China), and subsequently transferred to polyvinylidene fluoride (PVDF) membranes. After blocking with 5% skim milk in TBST for 1 hour at room temperature, membranes were incubated overnight at 4°C with primary antibodies against BRAFV600E (Cat. No. A25185, ABclonal, Wuhan, China) and β-Actin (Cat. No. #4967, Cell Signaling Technology, Danvers, MA, USA). After rigorous washing with TBST, membranes were incubated with horseradish peroxidase (HRP)-conjugated secondary antibodies (Cat. No. #7076 and #7074, Cell Signaling Technology) for 1 hour at room temperature. Protein bands were visualized using Pierce™ ECL Plus Substrate (Cat. No. 32132, Thermo Scientific) and detected with ChemiDoc XRS imaging system (Bio-Rad).

*Quantitative real-time PCR (qRT-PCR) array*

RNA extraction and qRT-PCR were conducted according to the manufacturer’s protocol. A custom qRT-PCR array (Wcgene Biotechnology Corporation, China) was designed to evaluate the expression of the BRAF25 genes, with *β*-actin and *GAPDH* as housekeeping genes. Cycle threshold (Ct) values were normalized to reference genes, and relative expression was calculated using the 2^−ΔΔCt method. Log2 fold-change values were computed for individual samples and group comparisons. The BRAF25 index was determined as the mean log2 fold-change of the BRAF25 gene expression values.

*Plasmid transfection*

Lipofectamine™ 3000 (Cat. No. L3000008, Thermo Scientific) was diluted 1:50 in Opti-MEM® (Cat. No. 31985062, Gibco). The BRAFV600E expression plasmid and empty vector control (Sunbio Medical Biotechnology) were diluted 1:100 in Opti-MEM, mixed with the transfection reagent, and incubated for 15 min at room temperature. Then, 200 µL of the transfection mix was added to each well. After 6 hours of incubation, the medium was replaced with fresh complete growth medium.

*CCK-8 assays*

1. *Cell viability assay*

Colon carcinoma cell lines (HT29, RKO, and COLO205) were seeded in 96-well plates at a density of 600 cells per well. Cells were treated with BCI, Vemurafenib, their combination, or DMSO (control). Cell viability was monitored daily for 6 consecutive days using the CCK-8 assay kit (Dojindo, Cat. No. CK04, Kumamoto, Japan), with absorbance measured at 450 nm using a FluoScan Ascent microplate reader.

1. *IC50 of BCI*

To evaluate the IC50 values of BCI, *BRAFV600E*-mutant (HT29, RKO, COLO205) and *BRAF*-WT (SW620, HCT115, HCT116) colon carcinoma cell lines were seeded at 1**×**10⁴ cells per well in 96-well plates. After 24 hours, cells were treated with an increasing gradient of BCI (ranging from 100 to 38400 nM). Following another 24-hour incubation, cell viability was assessed using the CCK-8 assay.

**Reference**

**1.** Sanchez-Vega F, Mina M, Armenia J, et al. Oncogenic Signaling Pathways in The Cancer Genome Atlas. *Cell.* 2018;173(2):321-337 e310.

**2.** Gai QJ, Fu Z, He J, et al. EPHA2 mediates PDGFA activity and functions together with PDGFRA as prognostic marker and therapeutic target in glioblastoma. *Signal Transduct Target Ther.* 2022;7(1):33.
